# Recent advances in sodium–sulfur batteries: cyclability and stability through electrolyte and separator engineering

**DOI:** 10.1039/d6ra06707g

**Published:** 2026-10-01

**Authors:** Akbota Kelgenbayeva, Aitolkyn Uali, Zhumabay Bakenov, Arailym Nurpeissova, Aliya Mukanova

**Affiliations:** a Institute of New Materials and Energy Technologies, Nazarbayev University Kabanbay Batyr Ave. 53 Astana 010000 Kazakhstan arailym.nurpeissova@nu.edu.kz aliya.mukanova@nu.edu.kz; b Institute of Batteries Kabanbay Batyr Ave. 53 Astana 010000 Kazakhstan uali_as@enu.kz; c Department of Chemistry, L.N. Gumilyov Eurasian National University Kazhymukan Street 13 Astana 010000 Kazakhstan; d Department of Chemical and Materials Engineering, School of Engineering, Nazarbayev University Kabanbay Batyr Ave. 53 Astana 010000 Kazakhstan; e National Academy of Sciences of Kazakhstan under the President of the Republic of Kazakhstan Kazakhstan

## Abstract

Metal–sulfur (M–S) batteries are emerging as promising solutions for future energy storage, thanks to their high theoretical energy density and the plentiful supply of sulfur. Among these, sodium–sulfur (Na–S) batteries are gaining popularity as affordable alternatives to lithium–sulfur (Li–S) systems because sodium is widely available and low-cost. However, developing effective room-temperature Na–S batteries for practical use remains challenging because sluggish reaction kinetics, sodium polysulfide dissolution, and shuttle effects together drive rapid capacity degradation. This comprehensive review examines the electrochemical mechanisms governing room-temperature Na–S batteries, with particular emphasis on the solubility and dissolution kinetics of sulfur species across diverse electrolyte systems. It points to the need to synergistically optimise electrolyte chemistry and calls for high-energy (high-concentration liquid, solid/quasi-solid and high-entropy) electrolytes, separator design, and solid-electrolyte interlayer architectures to prevent polysulfide migration and improve electrochemical stability, as well as for advanced experimental approaches (*in situ* and *operando* characterisation) and theoretical simulations and machine learning techniques. The review also highlights recent advances in Na–S battery prototypes to evaluate their practical performance and potential for technological adoption. Concluding with a perspective on Na–S batteries within the broader context of metal–sulfur energy storage systems, the review outlines critical directions for future research essential to realising their full practical potential.

## Abstract 2

Na–S batteries can achieve high stability and energy density when modified electrolytes, separators, and cathode binders suppress polysulfide dissolution and shuttling.

## Graphical abstract

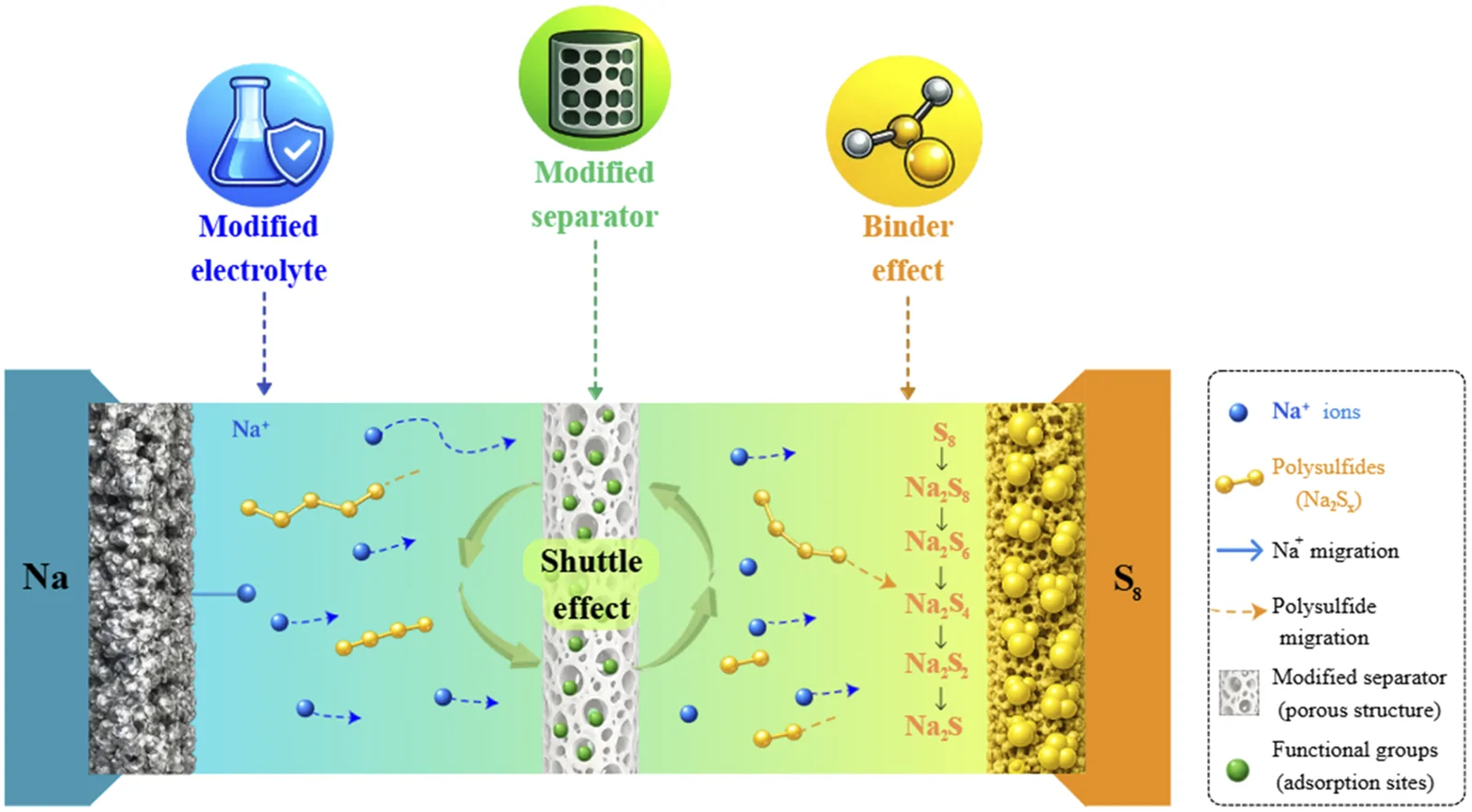

## 1 Introduction

Sodium–sulfur (Na–S) systems occupy a unique position as a cost-effective and scalable alternative to lithium–sulfur (Li–S) and other metal–sulfur (M–S) batteries. While lithium–sulfur batteries exhibit superior gravimetric energy density, sodium–sulfur batteries offer a more balanced combination of resource availability, economic feasibility, and electrochemical practicality. Together, these factors are critical for large-scale adoption. First, sodium is significantly more abundant than lithium, is widely available, and has relatively low raw-material costs (Fig. 1a). This can contribute to lower material costs and improved supply security for sodium–sulfur systems. For comparison, Li–S batteries represent a pivotal advancement in contemporary energy storage research.^1–4^ However, Li–S batteries suffer from capacity degradation associated with the polysulfide shuttle phenomenon in conventional liquid electrolytes.^5,6^ Nevertheless, despite their significant potential, challenges related to lithium resource scarcity persist. Limited quantities in the Earth's crust (approximately 20 ppm (ref. 7)) drive high production costs and supply-chain vulnerabilities. Additionally, limited geographic availability and rising lithium costs raise concerns about long-term sustainability. In contrast, sodium is approximately three orders of magnitude more abundant in nature (∼24 000 ppm (ref. 7)), widely available, and relatively low-cost.^7^ This significant difference in natural reserves makes sodium especially attractive for future battery technologies, particularly in stationary energy storage systems, where low cost and scalability are considered critical.^8^ These resource and cost considerations have prompted exploration of alkali-metal systems such as sodium and potassium.

**Fig. 1:**
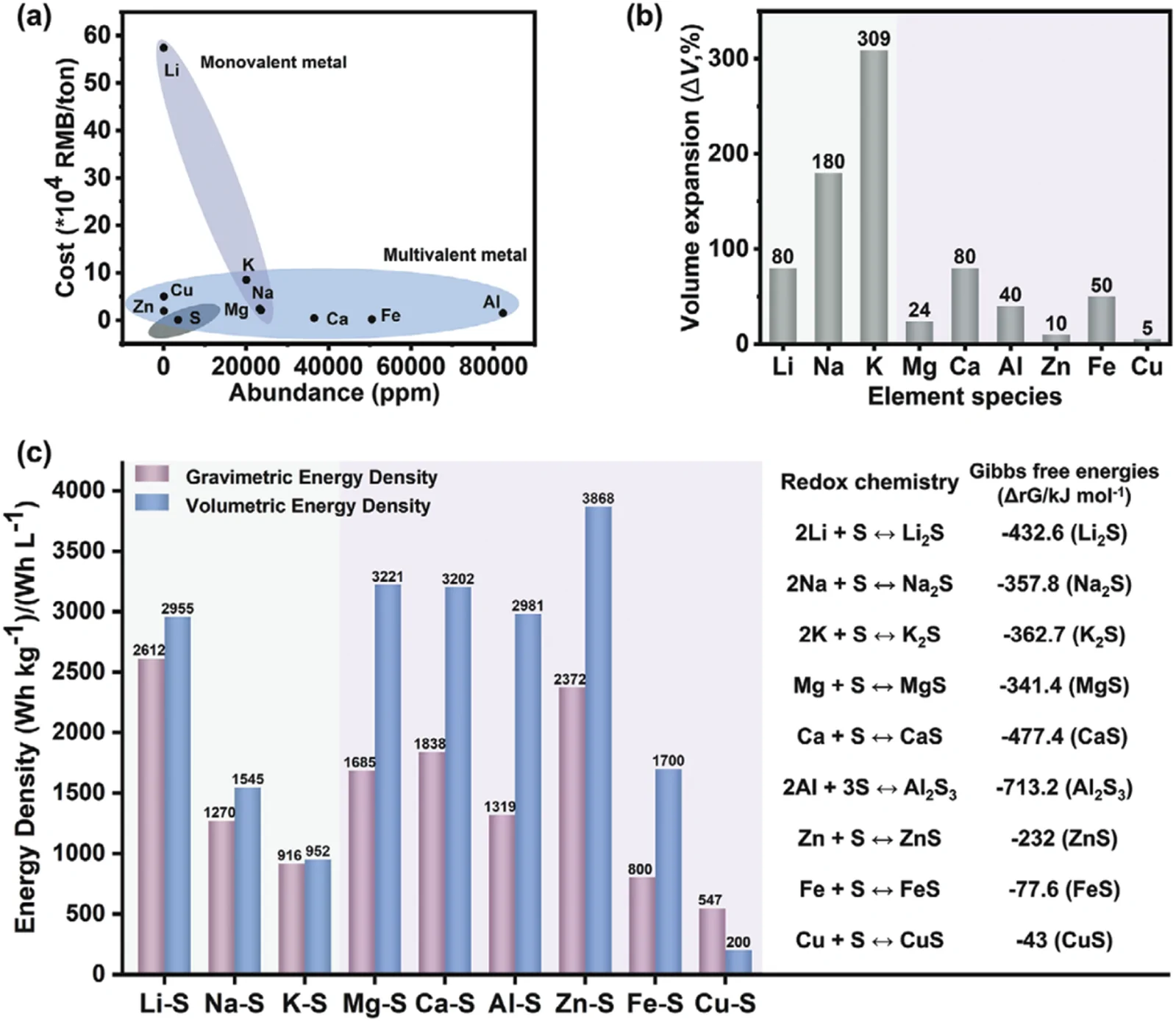
A comprehensive comparison between monovalent and multivalent M–S batteries, focusing on (a) cost and abundance, (b) volume expansion, and (c) gravimetric energy density (=*E*/*m*, where *E* and *m* are the theoretical energy and mass of the battery, respectively) and volumetric energy density (=*E*/*v* where *v* is the total volume of the battery), in conjunction with corresponding electrode reactions and Gibbs free energy. Reproduced from ref. 9 with permission from Wiley-VCH GmbH, copyright © 2024 under the CC BY-NC 4.0 license.

Secondly, volumetric expansion, a major challenge ultimately affecting a battery's lifespan and capacity, also highlights the practical advantage of sodium–sulfur systems, with the S-to-Na_2_S-associated volume expansion of ∼180% (Fig. 1b).^9^ Compared with Li–S, a benchmark with lower expansion (∼80%), Na–S batteries exhibit greater structural degradation.^10^ However, these problems are more manageable than those in potassium–sulfur (K–S) alternatives, which exhibit extreme ∼309% expansion and lead to severe mechanical degradation. Various battery design strategies, including porous carbon hosts, flexible conductive matrices, sulfur structures, carbon frameworks and specific separators, are employed to overcome these challenges in Na–S cells.^11^ From this perspective, the intermediate behaviour of Na–S batteries, which lies between monovalent systems, offers greater structural flexibility than heavier alkali-metal systems, while enabling performance optimisation through carbon matrix engineering and electrode design strategies.^7^

Beyond its abundance and relatively low production cost, sodium is a promising candidate for next-generation battery systems because of its distinctive electrochemical properties. As the second lightest alkali metal, sodium exhibits a redox potential of approximately –2.71 V *vs.* the SHE, only slightly lower than lithium's –3.04 V *vs.* the SHE.^12^ However, Na's larger ionic radius than Li^+^ results in sluggish solid-state diffusion and challenges electrode structural stability.^12^

Furthermore, energy density is also important, although it is relatively low (gravimetric energy density 1270 Wh kg^−1^). It is lower than that of Li–S (gravimetric energy density 2612 Wh kg^−1^) and other heavier alkali metal systems, but sufficient for stationary applications (Fig. 1c). Thermodynamically, the Na–S redox reaction remains highly favourable (∼−358 kJ mol^−1^), enabling efficient electrochemical conversion (Fig. 1с). As a result, Na–S batteries combine these properties with proven long service life and industrial maturity for large-scale energy storage infrastructure. This has led to the development of Na–S batteries, which are gaining increasing attention as a promising alternative to Li–S systems, particularly for large-scale energy storage applications. With the rapid rise of electric vehicles and the integration of renewable energy sources, the demand for affordable, scalable, and environmentally friendly energy storage solutions has become especially urgent.^7,13,14^ Although Li–S batteries have been extensively studied for over a decade and are moving towards commercial deployment, research on Na–S batteries has developed more gradually. Fig. 2 illustrates the trend in annual publications on Li–S and Na–S batteries from 1990 to 2026, based on a search of the Web of Science (WoS) database.

**Fig. 2:**
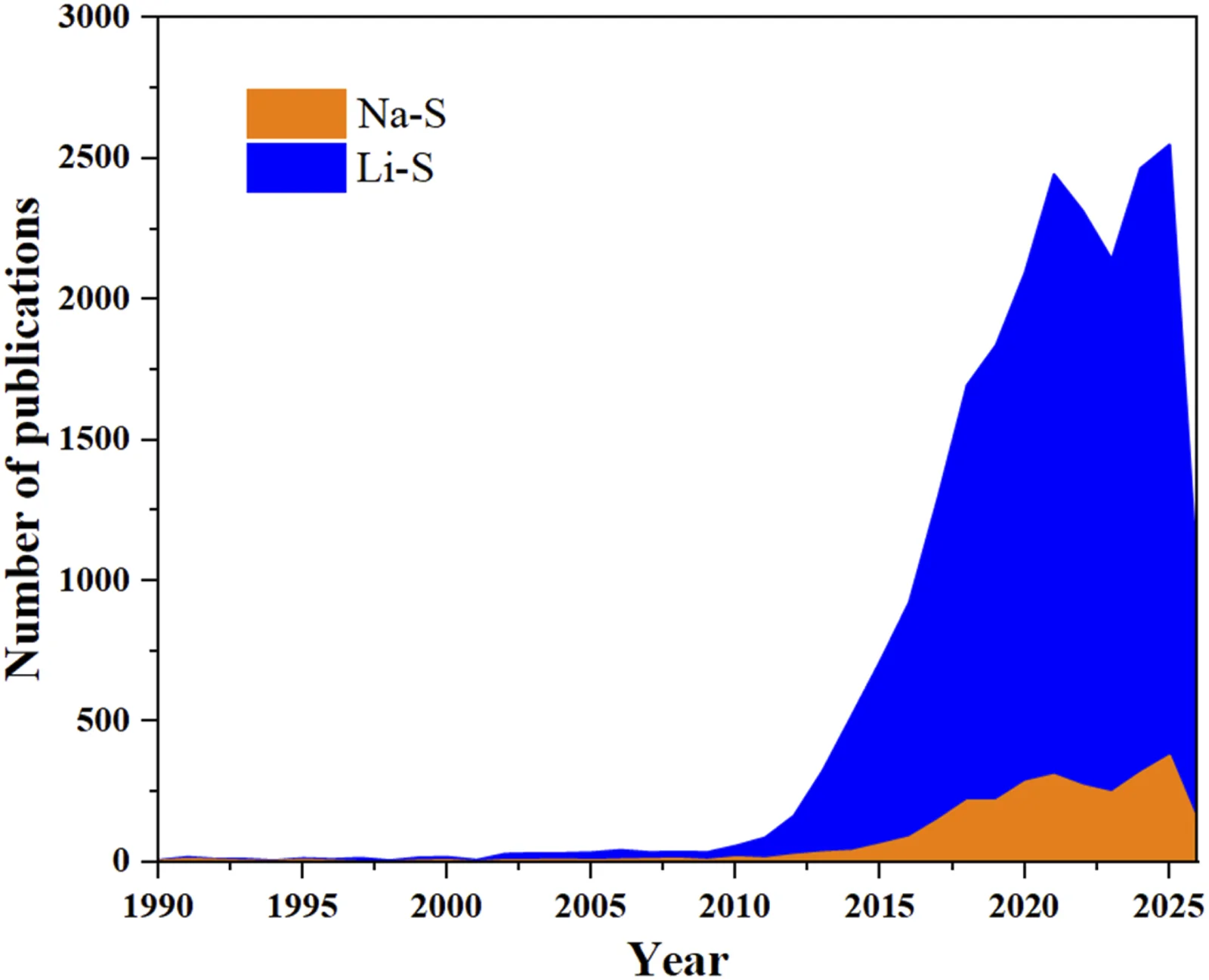
Annual number of publications on Li–S and Na–S batteries from 1990 to 2026, based on data^15^ retrieved from Web of Science (accessed on September 14, 2026).

The number of publications on both Li–S and Na–S batteries has increased since 2010, but considerably fewer studies have been published on Na–S batteries (Fig. 2). In 2026, 812 publications were recorded for Li–S batteries, compared with 132 for Na–S batteries. Despite the increasing number of studies, Na–S batteries remain comparatively less investigated, indicating that further fundamental and technological research is still needed. In this context, systematic reviews of recent progress in Na–S systems appear particularly timely.

Some provide a general overview of all sodium-based battery systems; others focus on the thermodynamics and kinetics and the atom-scale mechanism behind the Na–S battery's performance.^16^ Another group of review papers in this area has raised questions about cathode and binder design,^17–20^ separators^21,22^ and electrolytes.^23,24^ More recently, Song *et al.*^25^ reviewed electrolyte additives and separator strategies specifically for room-temperature Na–S batteries. In ref. 8, Na–S systems are discussed generally, with a specific focus on recycling. Recent advances^26–29^ in room-temperature (RT) Na–S systems have also been reported, further expanding their application range. Although these reviews cover several aspects of RT Na–S batteries, their scope and organisation differ depending on the specific problem or cell component considered. Building on these studies, the present review brings these aspects together by linking the fundamental processes of sulfur dissolution, polysulfide formation, and transport to the electrolyte, separator/interlayer, and cathode strategies used to regulate them, while also considering Na–S chemistry within the broader framework of M–S energy storage. Specifically, Section 1 deconstructs the fundamental electrochemical mechanisms governing sulfur conversion chemistry, highlighting challenges in mass-transport kinetics and the diffusion of sulfur species, with a focus on sulfur solubility and polysulfide transport dynamics (Section 2). The subsequent section critically discusses various electrolyte design strategies, including high-performance (localised) high-concentration liquid electrolytes, high-entropy electrolytes, and solid- and quasi-solid-state electrolytes, along with their limitations and future research directions. Section 4 discusses separator and interlayer engineering to control NaPS transport while maintaining Na^+^ transport. Ion-selective transport, physical confinement, adsorption, and catalytic functions are considered. Section 5 discusses research-scale Na–S cell prototypes, emphasizing cathode designs for sulfur confinement and polysulfide regulation, together with electrochemical performance and practical cell parameters, including sulfur loading, areal capacity, and electrolyte-to-sulfur (*E*/*S*) ratio, where these data are available. Section 6 compares RT Na–S batteries with other metal–sulfur systems, then discusses remaining challenges and future research directions.

## 2 Electrochemical mechanisms and challenges of Na–S batteries

The Na–S battery consists of a sulfur cathode, a sodium metal anode, a separator, and a sodium-ion-conducting electrolyte, and operates *via* a series of reactions that begin with the ring-opening of β-S_8_.^9^ This reaction leads to the formation of long-chain sodium polysulfides (Na_2_S_*x*_, where 4 ≤ *x*≤ 8), followed by the generation of short-chain sodium polysulfides (Na_2_S_*x*_, where 1 ≤ *x*≤ 4). As shown in Fig. 3, the discharge curve of the room-temperature Na–S battery can be divided into four distinct stages. In the first stage, the solid elemental sulfur β-S_8_ is reduced to a soluble long-chain Na polysulfide (Na_2_S_8_), which corresponds to the plateau at 2.2 V. Stage II then corresponds to the sloping curve in the voltage region from 2.2 V to 1.65 V, in which Na_2_S_8_ converts in solution to Na_2_S_4_. In turn, the third stage corresponds to the 1.65 V plateau, where the liquid Na_2_S_4_ reduces to insoluble short-chain Na polysulfides, *i.e.*, Na_2_S_3_, Na_2_S_2_, and Na_2_S. Stage IV corresponds to the sloping curve between 1.65 V and 1.2 V, where a solid-to-solid transition from Na_2_S_2_ to Na_2_S occurs.^12^

**Fig. 3:**
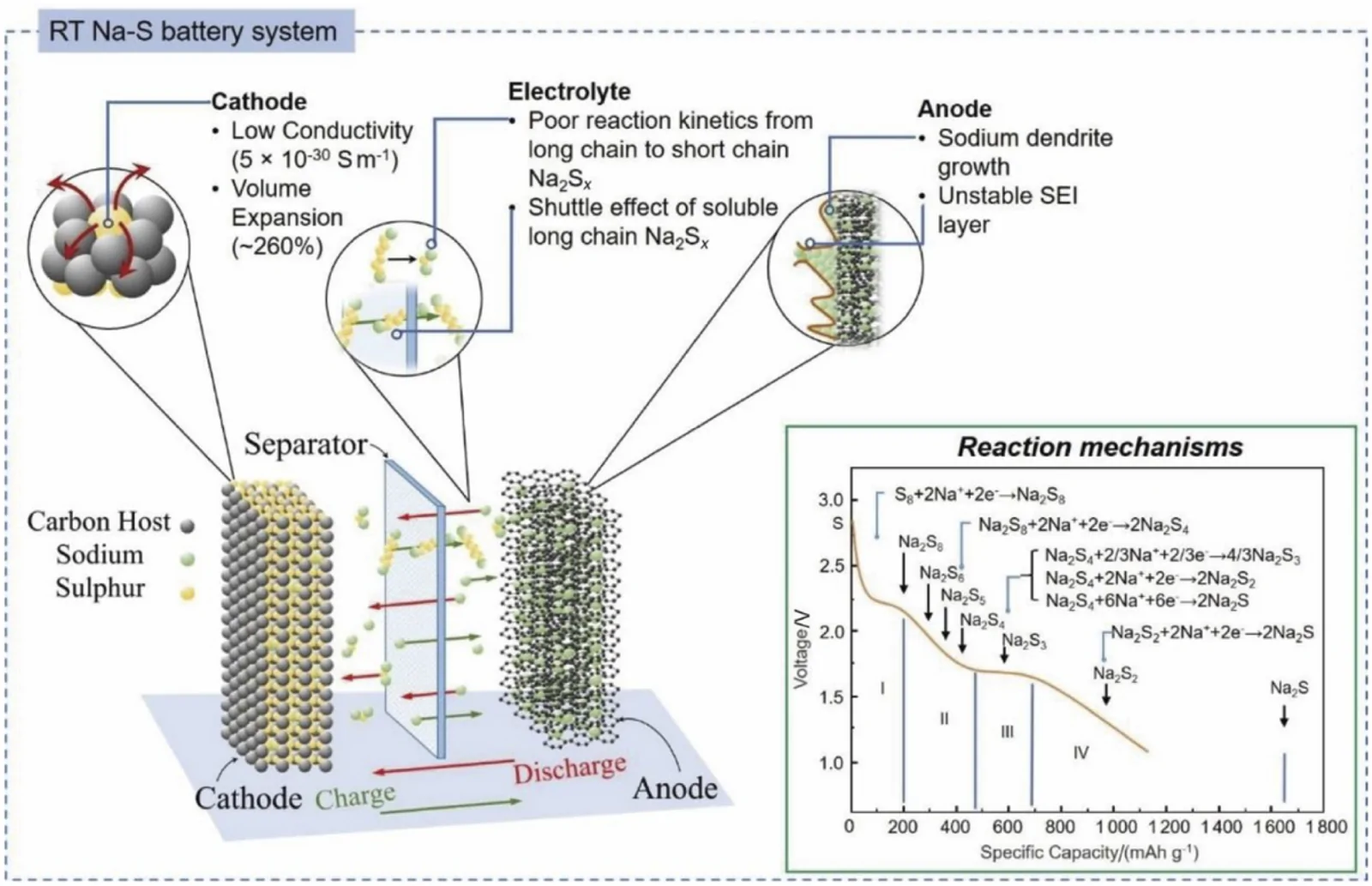
Structure and challenges of RT Na–S batteries. Inset: reaction mechanisms of RT Na–S batteries. Reproduced from ref. 30 with permission from Springer Nature, copyright © 2023 under the CC BY 4.0 license. (Note that the ∼260% volume expansion indicated in the reproduced Fig. 3 is the value reported in ref. 30; in the present review, ∼180% is adopted for the S-to-Na_2_S volume expansion following ref. 9).

As the perspectives for M–S battery development,^9^ including Na–S batteries, evolve, it is essential to address key aspects, such as an in-depth understanding of sulfur conversion chemistry, material design or structural optimisation for sulfur cathodes, potential directions for metal anodes in sulfur-rich environments, and novel approaches to electrolyte formulations or configuration designs, while taking into account all the challenges in this area.

One of the key challenges in all M–S batteries is sulfur's extremely low electronic conductivity (5.13 × 10^−28^ S m^−1^).^31^ As a consequence, electron transport within the cathode becomes inefficient, reflected in low active-material utilisation, poorer performance at higher current densities, and increased cell internal resistance. The accumulation of solid discharge products (Na_2_S) formed during electrochemical conversion also contributes to overall resistance.^16^ Moreover, sodium reacts poorly with solid sulfur in liquid electrolytes, further limiting redox-process efficiency. During cycling, a solid–liquid–solid phase transformation occurs, causing substantial volumetric expansion of the cathode (up to 180% upon full discharge to Na_2_S), leading to active-material delamination, structural collapse, and loss of electrical contact.^9,19^

It is known that Li_2_S is the only stable solid phase in the Li–S phase diagram (Fig. 4a).^32^ In contrast, the Na–S system includes Na_2_S, Na_2_S_2_, Na_2_S_4_, and Na_2_S_5_ as stable at room temperature (Fig. 4b). Additionally, larger Na atoms lead to sluggish kinetics but also facilitate easier electron loss, making Na more reducing. Based on this, and to address questions about sulfur reaction intermediates, conversion steps during charge/discharge, and kinetic influence,^32^ conducted a more detailed examination of the intrinsic thermodynamic and kinetics-controlled electrochemical behaviours of sulfur reactions in all-solid-state batteries (ASSBs) (Fig. 5).

**Fig. 4:**
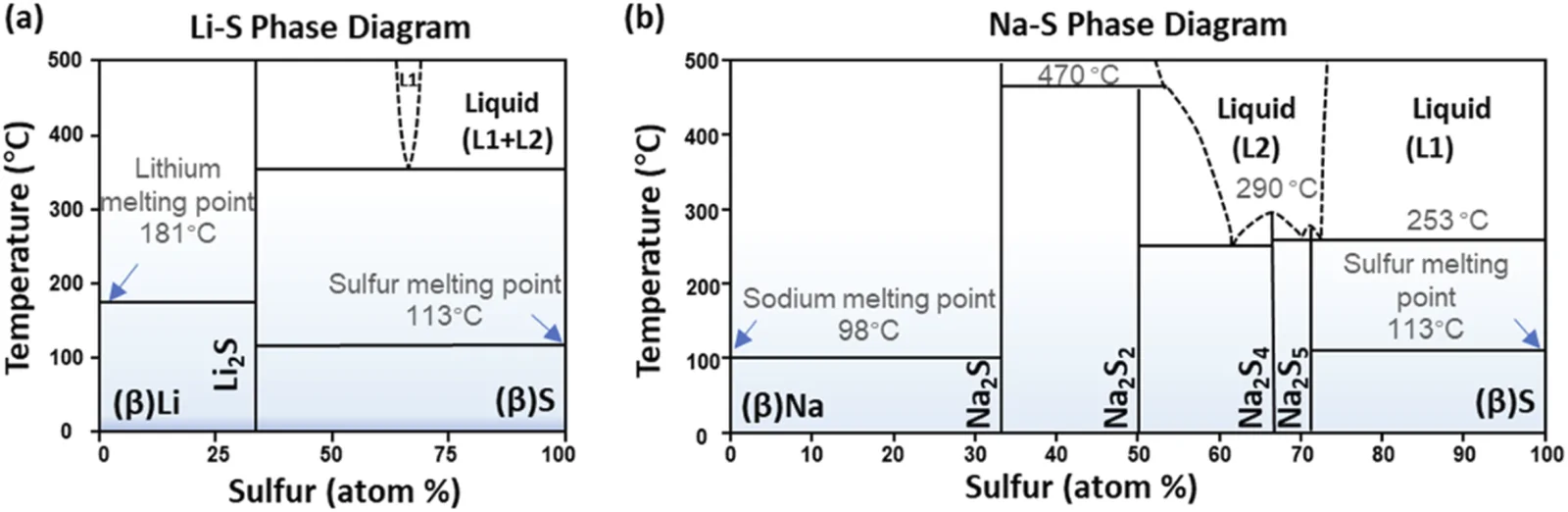
Phase diagrams of (a) Li–S and (b) Na–S systems. Reproduced from ref. 32 with permission from the Royal Society of Chemistry, copyright © 2024 under the CC BY 3.0 license.

**Fig. 5:**
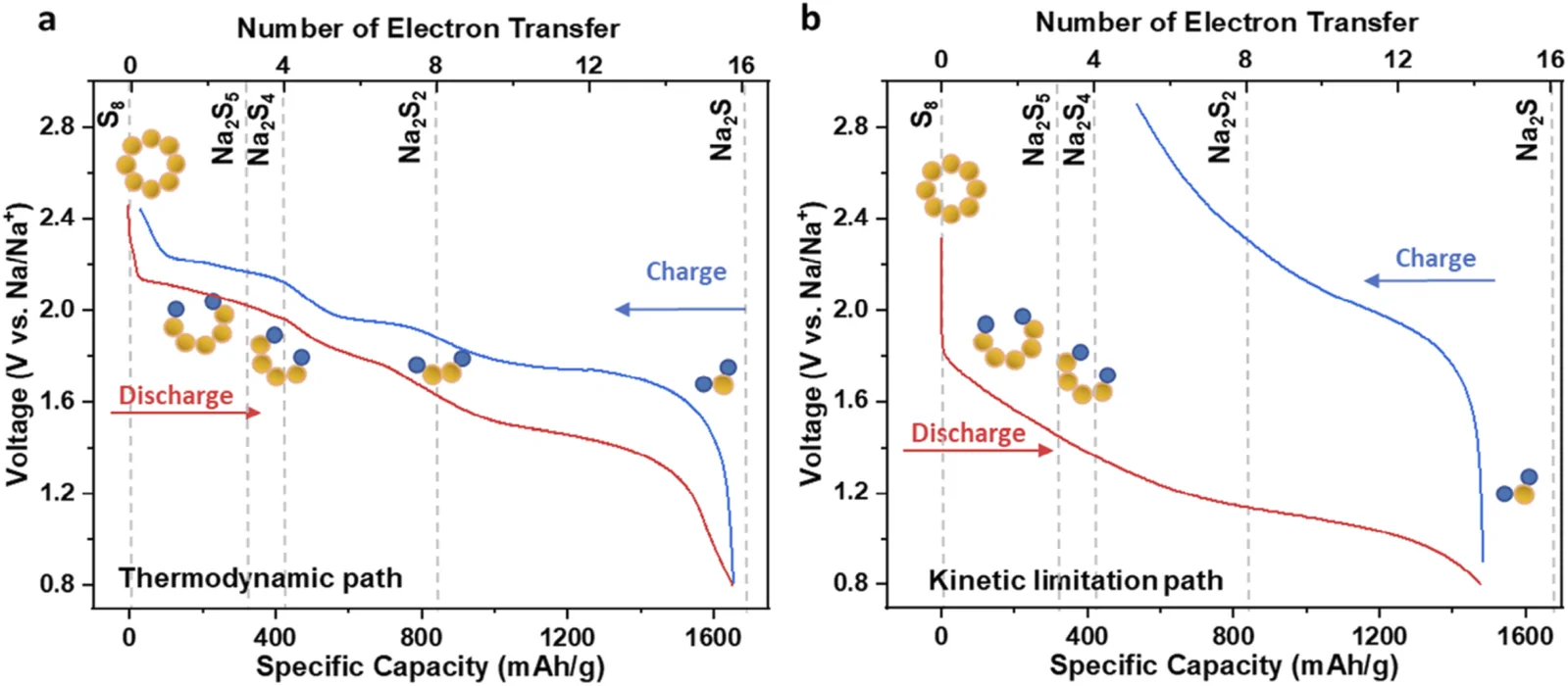
Reaction pathways under thermodynamic and kinetic effects. The voltage profiles of the ASSBs operated under (a) the thermodynamic path and (b) the kinetic limitation path. Reproduced from ref. 32 with permission from the Royal Society of Chemistry, copyright © 2024 under the CC BY 3.0 license.

As shown in Fig. 5a, the thermodynamic pathway of the sulfur cathode features three plateau regions.^32^ In the first plateau, S_8_ initially reduces to Na_2_S_5_ and Na_2_S_4_, then transforms into Na_2_S_2_ in the second plateau. In the third plateau, reduction continues to Na_2_S. However, as shown in Fig. 5b, at low temperatures (≤60 °C) and standard rates (≥C/20), the kinetics become sluggish, shifting the sulfur-reduction pathway and increasing overpotential. In the kinetic scenario, two plateaus occur because the Na_2_S_2_ phase is skipped, leading to transitions from Na_2_S to Na_2_S_4_ during charging and from Na_2_S_4_ to Na_2_S during discharging. Furthermore, incomplete conversion of Na_2_S_4_ to S_8_ during charging, due to slow kinetics at room temperature, results in a low first-cycle charging capacity, which subsequently affects capacity in subsequent cycles. A deeper atomic-scale comparison of mechanisms and kinetics in Li–S and Na–S batteries reveals that the discharge rate-limiting step is faster in Li–S batteries.^26^ In contrast, the decomposition kinetics are quicker in Na–S batteries.

Another major challenge arises from the formation of soluble sodium polysulfides (Na_2_S_*n*_, 4 ≤ *n*≤ 8) during discharge.^21^ These higher-order compounds are often electrochemically inactive during charging, leading to the accumulation of “dead sulfur” on the anode, loss of active material, and increased irreversible capacity.^33^ This phenomenon, known as the shuttle effect, involves the repeated migration and redox cycling of polysulfide species between the cathode and anode.^34^ In Na–S batteries, this effect is more severe than in Li–S systems for several reasons. First, the larger ionic radius of Na^+^ intensifies cathode structural deformation.^35^ Second, sodium's lower electronegativity weakens its interactions with sulfur anions, reducing interfacial stability and slowing reaction kinetics.^35^ This promotes faster accumulation of soluble intermediates and accelerates capacity decay. In addition, progressive electrode passivation during cycling increases impedance—especially during deep discharge, when solid Na_2_S_2_ formation limits further electrochemical reactions.^36^ These cumulative effects make developing efficient room-temperature Na–S batteries particularly challenging. Note that despite the significant advantages of Na–S batteries—including high theoretical energy density and the abundance of raw materials—their practical application remains limited. The main reasons include unstable cycle life, performance degradation, and serious safety concerns.^37^

Regarding the RT Na–S batteries, recent studies on the solubility and dissolution kinetics of sulfur and sulfides in electrolyte solvents reveal several critical challenges:^27,38^ low solubility and slow kinetics of Na_2_S_2_/Na_2_S, limited quantitative solubility and diffusion data, the polysulfide shuttle effect, complex multi-step redox chemistry, electrolyte instability and side reactions, solid-state precipitation and passivation, absence of tailored cathode hosts for Na chemistry, and poorly modelled dissolution transport-redox coupling. Altogether, these findings highlight the need for a systems-level engineering approach, including optimising cathode architecture, suppressing the shuttle effect, and stabilising interfacial chemistry. This difference clearly highlights the significant knowledge gap that must be addressed to fully develop Na–S technologies. Given their potential advantages—high theoretical energy density, low cost, and resource availability—intensified research efforts in this area are essential.^12^ Further advances in cathode materials, electrolytes, and interface engineering could open new horizons for developing highly efficient Na–S batteries.

## 3 Solubility and dissolution kinetics of sulfur and sulfides in electrolyte solvents for sodium–sulfur batteries

In general, the S dissolution kinetics at the solid–liquid interface can be expressed as (eqn (1)):^39^1
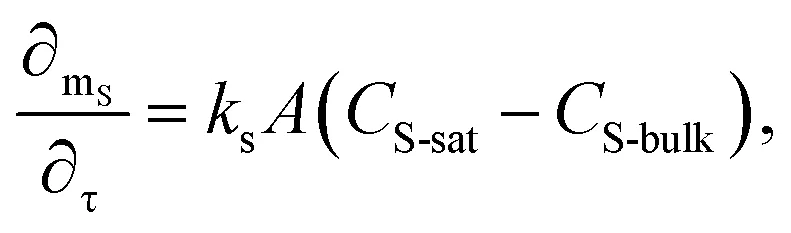
in which 
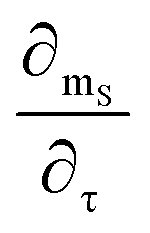
 is the rate of the solute dissolution (mol s^−1^); *C*_S-sat_ and *C*_S-bulk_ are the saturation and bulk concentrations of solute in the solution (mol m^−3^); *k*_s_ is the mass transfer coefficient (m s^−1^) of solute in the solution; *A* is the area of a distinct plane solid–liquid interface (m^2^).

At the equilibrium condition, a sulfide solution of Na_2_S_*x*_ can be represented as follows [chem eqn (2)]:^39^

2 Na_2_S_*x*_ ↔ 2Na^+^ + S_*x*_^2−^

The solubility product *K*_sp_ is given as a function of the saturation concentration *C*_S-sat_ (in S mol L^−1^) by the relation (eqn (3)):^39^3
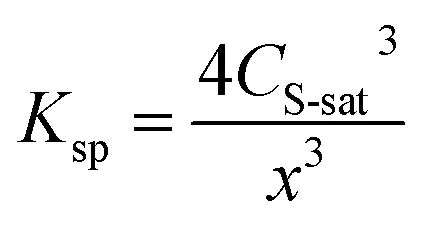


According to Fick's law, the solvent goes through diffusion into the solid phase (eqn (4)):^39^4
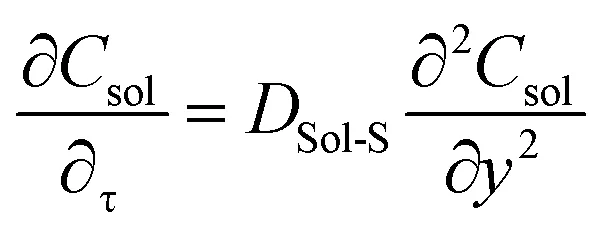
where *C*_sol_ is the solvent concentration in the solid (mol m^−3^) and *D*_Sol-S_ is the solvent's diffusion coefficient in the solid phase (m^2^ s^−1^).

Fickian diffusion describes concentration-driven transport in Na–S batteries and serves as a useful first-order interpretation. However, an operating cell is fundamentally a coupled reaction-transport system in which charged Na^+^ ions and polysulfide species undergo both diffusion and electric-field-driven migration, while ion association, solvation, electrochemical conversion and Na_2_S_2_/Na_2_S precipitation continuously modify species mobility and concentration. Consequently, apparent Fickian diffusivities often reflect reaction and phase-transition kinetics rather than intrinsic molecular diffusion. Ji *et al.*^32^ observed pronounced diffusivity minima during galvanostatic intermittent titration technique (GITT) measurements in two-phase regions, identifying the reaction sequence S_8_ → Na_2_S_5_/Na_2_S_4_ → Na_2_S_2_ → Na_2_S under sufficiently slow or elevated-temperature conditions. Recent studies further highlight the importance of coupled transport. In ref. 40, polysulfide solvation controls interfacial chemistry and mass transport within the electric double layer, while^41^ identified Na^+^ depletion and Na_2_S precipitation as rate-limiting processes. Therefore, Nernst–Planck models incorporate both diffusion and electromigration, while complete concentrated-solution or reaction-transport descriptions additionally capture non-ideal ion association, interfacial reactions, polysulfide reactions, and precipitation^42^ – offering a more suitable framework for predictive modelling of practical Na–S cells.

In Na–S batteries, elemental sulfur is typically embedded in a carbon host and can be electrochemically reduced. During discharge, this forms various sodium polysulfides (Na_2_S_*x*_) (Fig. 3). With a liquid electrolyte, higher-order soluble Na_2_S_*x*_ intermediates undergo rapid liquid-phase redox reactions, facilitating stable long-range migration from the cathode to the sodium anode,^17^ whereas short-chain species, Na_2_S_2_ and Na_2_S, suffer from both thermodynamic and kinetic limitations. Thermodynamically, Na_2_S_2_ and Na_2_S exhibit extremely low equilibrium solubility in conventional electrolytes.^43^ This low solubility restricts active-material dissolution, hindering phase-boundary contact and limiting the intrinsic reversibility of redox reactions.^44^ Thus, the solid-state conversion of these species is sluggish. The slow dissolution rates and sluggish interfacial electron-transfer kinetics of Na_2_S and Na_2_S_2_ impede reaction completion, leading to premature precipitation on active cathode surfaces.^40^ The resulting insulating layers passivate the electrode, increase polarisation, and cause progressive capacity degradation. To support this, in ref. 45, *ex situ* UV-vis and *in situ* Raman spectroscopic mapping of the evolution of NaPS species during the charging and discharging of Na–S cells shows that Na_2_S_8_, Na_2_S_4_, and Na_2_S_2_ are key intermediates, with S_8_ as the starting product and Na_2_S as the final product. However, the intermediate Na_2_S_6_ does not contribute to the conversion mechanism. Because Na_2_S_6_ forms *via* the comproportionation reaction between Na_2_S_8_ and Na_2_S_4_, it contributes to active-material loss during charging. Moreover, the study points out that Na_2_S_6_ contributes to active-material loss through dissociation that forms S_3_˙^−^ radicals. The disproportionation of Na_2_S_4_ can also form the radical S_3_˙^−^. These processes are in equilibrium within the electrolyte and typically result in active-material loss to it.

Furthermore, limited data exist on the structural and transport properties of Na–S battery electrolytes – namely the saturation solubility of polysulfide species in the solvent (*C*_S-sat_), the dissolution/mass-transfer coefficient (*k*_s_), and the diffusion coefficient of the solvent into the solid sulfur/sulfide phase (*D*_Sol-S_). Table 1 synthesises these core physicochemical characteristics of sodium polysulfide (N_a2_S_*x*_) species, focusing on their solubility, mass-transfer kinetics and diffusion dynamics. The thermodynamic stability and solubility of NaPSs in glyme-based solvent systems depend strongly on the polysulfide chain length. High-order, long-chain polysulfides (Na_2_S_*x*_, 4 ≤ *x*≤ 8) exhibit high solubility in conventional ether media, such as TEGDME and binary mixtures. This high solubility enables long-chain species to act as soluble catholytes, but it also accelerates parasitic shuttle phenomena. Conversely, as reduction proceeds towards short-chain species (*x* ≤ 3), the systems experience a non-linear drop in solubility. Intermediate species such as Na_2_S_3_ show significantly reduced concentrations, while the final solid discharge products (Na_2_S_2_, Na_2_S) are insoluble.

**Table 1:** Quantitative dissolution, solubility and diffusion data for sodium polysulfides relevant to RT Na–S batteries^a^

Na_2_S_*x*_ species	Solvent/electrolyte medium	Electrolyte/salt, concentration	Temperature, °C	Saturation solubility^b^, *C*_S-sat_, S-mol L^−1^	Dissolution/mass-transfer coefficient, *k*_s_, m s^−1^	Diffusion coefficient of the solvent into solid sulfur/sulfide phase, *D*_Sol-S_, m^2^ s^−1^	Method used for diffusion coefficient determination	Note	Ref.
Long-chain Na_2_S_*x*_, 4 ≤ *x*≤ 8	TEGDME-based catholyte	Liquid sodium-polysulfide catholyte	RT	n.r.	n.r.	n.r.	Electrochemical investigation of polysulfide transport/shuttling. No diffusion measurement	NaPS can function as soluble catholytes	47
Na_2_S_8_	TEGDME	Electrolyte environment	RT	10.56	n.r.	n.r.	Equilibrium solubility experiment and visual/species identification in the compartmentalized electrochemical cell. No diffusion measurement	Very high value of solubility compared to later measurements	48
Na_2_S_6_	TEGDME			7.14	n.r.	n.r.		Higher than later reported for measured RT range	
Na_2_S_5_	TEGDME			2.14	n.r.	n.r.		Chain-length-dependent NaPS solubility	
Na_2_S_4_	TEGDME			1.76	n.r.	n.r.		Closest of the high-order values to the later measurements	
Na_2_S_3_	TEGDME			0.039	n.r.	n.r.		Consistent with later measurements.	
Na_2_S	TEGDME			<0.0067	n.r.	n.r.		Extremely low solubility.	
Dissolved NaPSs, primarily long-chain species	TEGDME	Na–S electrolyte	RT	n.r.	n.r.	n.r.	H-type diffusion cell (H-cell)/polysulfide permeation test. Not quantitative determination of diffusion coefficient	Suppression of Na_2_S_8_ migration in TEGDME by highly N,S-doped nanoporous carbon	49
S_8_	DOL : DME, 1 : 1 (v/v)	Salt-free	21	0.11 ± 0.05	(3.5 ± 0.1) × 10^−3^	(9.7 ± 4.6) × 10^−12^	Time-resolved dissolution experiment coupled with Fickian diffusion/mass transfer model fitting	—	39
Na_2_S_8_	DOL : DME, 1 : 1 (v/v)			0.50 ± 0.10	n.r.	n.r.		Long-chain NaPS is appreciably soluble	
Na_2_S_6_	DOL : DME, 1 : 1 (v/v)			0.50 ± 0.10	n.r.	n.r.		The same as for Na_2_S_8_ under identical conditions	
Na_2_S_4_	DOL : DME, 1 : 1 (v/v)			0.50 ± 0.10	n.r.	n.r.		Relatively soluble.	
Na_2_S_3_	DOL : DME, 1 : 1 (v/v)			0.150 ± 0.010	(0.9 ± 0.2) × 10^−3^	(5.3 ± 1.6) × 10^−9^		Considerably lower solubility than long-chain NaPS, but dissolution kinetics is measurable	
Na_2_S_2_	DOL : DME, 1 : 1 (v/v)			0.013 ± 0.002	(4.5 ± 1.6) × 10^−3^	(3.8 ± 3.0) × 10^−10^		Strong precipitation.	
Na_2_S	DOL : DME, 1 : 1 (v/v)			0.005 ± 0.001	(21.9 ± 9.6) × 10^−3^	(8.0 ± 7.9) × 10^−10^		A strongly precipitating terminal discharge product	
S_8_	TEGDME	Salt-free	21	0.07 ± 0.05	(0.4 ± 0.1) × 10^−3^	(2.2 ± 1.0) × 10^−10^	Time-resolved dissolution experiment coupled with Fickian diffusion/mass transfer model fitting	—	39
Na_2_S_8_	TEGDME			1.25 ± 0.10	n.r.	n.r.		TEGDME solvates long-chain NaPSs stronger than DOL : DME.	
Na_2_S_6_	TEGDME			1.25 ± 0.10	n.r.	n.r.		High long-chain NaPS solubility	
Na_2_S_4_	TEGDME			1.25 ± 0.10	n.r.	n.r.		Long-chain/intermediate NaPS remain highly soluble	
Na_2_S_3_	TEGDME			0.039 ± 0.010	(8.8 ± 5.4) × 10^−3^	(1.4 ± 0.9) × 10^−7^		Swollen semisolid Na_2_S_3_ phase.	
Na_2_S_2_	TEGDME			0.013 ± 0.002	(10.5 ± 1.2) × 10^−3^	(1.8 ± 0.4) × 10^−10^		Low solubility despite fast dissolution kinetics	
Na_2_S	TEGDME			0.0057 ± 0.001	(17.6 ± 6.1) × 10^−3^	(1.9 ± 1.3) × 10^−9^		Terminal Na_2_S remains effectively insoluble	
Na_2_S_*x*_ species generated *operando*	TEGDME	1 M NaCF_3_SO_3_ in TEGDME	RT	n.r.	n.r.	n.r.	Spectroscopic (*in situ* Raman and *ex situ* UV-vis spectroscopy) polysulfide identification. No diffusion measurement	Directly resolves polysulfide evolution and distribution	45

a n.r. is for “not reported”.

b Concentration on a sulfur-atom-equivalent basis, not necessarily mol L^−1^ of Na_2_S_*x*_ molecules.

In species-transfer modelling, the diffusion coefficient, *D*_Sol-S_, quantifies solvent transport through the solid layer.^39^ Whereas elemental sulfur (S_8_) remains largely undissolved during battery cycling, cation transport and sulfide reactions can occur either in the solid phase or in a semi-solid phase where some solvent could diffuse.^39^ In Li–S systems, the Li^+^ diffusion coefficient varies with polysulfide (PS) specification and concentration.^46^ Although shorter polysulfides diffuse faster than longer chains, this kinetic distinction disappears at higher PS concentrations (>0.5 M). Molecular dynamics simulations of the electrolyte's structural and transport characteristics indicate that the binding between PS and Li^+^ ions appears excessively strong, leading to an overestimation of ion-pair formation.^46^ Furthermore, adding 1 M LiTFSI to the system reduces diffusion by about 50% at high PS concentrations, while introducing LiTFSI into dilute PS solutions increases the number of dissociated Li^+^ and leads to higher conductivity. As previously mentioned, data on diffusion and other transport properties for Na–S batteries remain limited (Table 1).

Generally, long-chain polysulfides are more soluble in polar liquid solvents, while data on diffusion coefficients are limited (Table 1). Conversely, short-chain polysulfides demonstrate limited solubility. In polar ether solvents and electrolyte systems, low-order NaPSs exhibit relatively consistent diffusion coefficients on the order of ≈10^−9^ – 10^−10^ m^2^ s^−1^, with a higher diffusion coefficient for Na_2_S_3_ in a TEGDME-based electrolyte (1.4 × 10^−7^ m^2^ s^−1^). Accelerating diffusion typically requires high-dielectric-constant, low-viscosity solvents to enhance the mobility of both Na^+^ and polysulfide species.^23^ Furthermore, diffusivity follows the Arrhenius relationship: rising temperatures decrease viscosity and increase ionic mobility, leading to an exponential rise in polysulfide diffusivity.^50^ Additionally, additives such as MoTe_2_ nanosheets can enhance catalytic performance in sulfur redox reactions and facilitate Na^+^ ion diffusion even under lean electrolyte conditions with a high sulfur loading of 3.5 mg cm^−2^.^51^ This configuration achieves a high discharge capacity of 1081 mAh g^−1^, with a minimal capacity fading rate of 0.05% per cycle over 350 cycles at 0.1C. While polysulfide diffusivity under different environmental conditions is well studied, strategies to regulate diffusion kinetics effectively remain understudied, especially in tailored electrolytes and composite cathode matrices. Unfavourable features of Na–S batteries, including the severe shuttle effect arising from the dissolution of high-order sodium polysulfides in electrolyte solvents and poor conductivity, hinder their practical use.^18^ Mitigating the shuttle effect while enhancing the electronic conductivity of Na–S batteries is crucial to achieving the desired performance of next-generation Na–S batteries.^52^ Because the electrochemical performance, cycle life, and stability of Na–S batteries are strongly governed by sulfur solubility, dissolution kinetics, and reduced sulfide species, these factors are vital to battery function. Furthermore, integrating emerging technologies such as machine learning and artificial intelligence could revolutionise theoretical and practical battery design.

## 4 Electrolytes for the Na–S battery architecture

### 4.1 Liquid electrolytes

Consider tailoring electrolyte chemistry from distinct mechanistic perspectives. First, polysulfide solubility, determined by solvent donor strength, dielectric environment, salt concentration, and Na^+^-polysulfide association, governs the extent of Na_2_S_*x*_ dissolution and shuttling. Second, the sulfur-conversion pathway describes which chemical and electrochemical intermediates mediate the S_8_-to-Na_2_S conversion, governed by thermodynamics and kinetics.^53^ Third, Na-metal stability involves electrolyte reduction, Na plating/stripping uniformity, and dendrite formation and chemical attack by crossover NaPS; primarily influenced by Na^+^ solvation/desolvation and interfacial chemistry rather than polysulfide solubility alone. Fourth, SEI/CEI formation arises from electrolyte decomposition. Finally, transport encompasses Na^+^ conductivity, transference/desolvation, and diffusion at interfaces: increasing salt concentration or reducing free solvent may enhance interfacial stability and lower NaPS mobility but could raise viscosity and decrease conductivity; thus, transport must be optimised independently. Overall, electrolyte chemistry influences these five interconnected links, and parameter changes can affect multiple aspects simultaneously.

An analysis of representative liquid electrolyte classes reveals a clear paradigm shift in battery chemistry: a transition from simple ether-solvating systems to weakly coordinating, localised systems and asymmetric and high-entropy solvation architectures. In Na–S batteries operating at room temperature, similar to liquid electrolytes used in Li–S batteries, ether- and amide-based electrolytes such as tetraethylene glycol dimethyl ether (TEGDME) and *N*,*N*-dimethylacetamide (DMA) can be used.^24,54^ In such electrolyte systems, sulfur is reversibly reduced to highly soluble long-chain NaPSs as intermediates and is eventually converted to solid Na_2_S_2_ and Na_2_S during discharge.^40^ These NPSs migrate uncontrollably to the Na anode, causing active-material loss and severe deterioration of interphase boundaries, thereby significantly impairing reversibility and cycle life.^50,52^ As seen from Table 2, standard ether systems deliver acceptable initial discharge capacities, often exceeding 1000 mAh g^−1^ at low current rates. However, their long-term cycling stability is severely compromised, experiencing a dramatic capacity drop-off within 10–300 cycles. In ref. 48, the optimised Na–S cell demonstrates a high initial capacity of 1070 mAh g^−1^ during the first discharge, which, over only 37 cycles, is maintained at 782 mAh g^−1^. The cell showed robust rate performance, delivering 569 mAh g^−1^ at 0.5C, while coulombic efficiency approached 100% after the 5th cycle. As highlighted, the extent of polysulfide dissolution correlates directly with the molar concentration of sulfur.^48^ To mitigate polysulfide migration, carbonate (*e.g.*, propylene carbonate (PC), dimethyl carbonate (DMC), and fluoroethylene carbonate (FEC))-based/added liquid electrolytes are also used as an alternative to ether-based electrolytes. The state of sulfur plays a pivotal role in its conversion in a carbonate-based electrolyte.^55^ During initial discharge, the crystalline sulfur outside the pores converts to NaPSs. This involves an irreversible reaction with the electrolyte. Meanwhile, amorphous sulfur confined within the pore structure undergoes a two-step solid-state reaction, enhancing the system's reversible capacity. Appropriate pore architecture in the cathode or cathode host is crucial for creating the necessary chemomechanical stress within the confined space, which significantly impacts sulfur conversion kinetics in Na–S batteries at room temperature. In this context, the role of the carbonate electrolyte in transforming sulfur into another chemical form is particularly emphasised: its ability to accommodate the maximum number of reversible sulfur molecules while ensuring rapid Na^+^ ion transfer.

**Table 2:** Comparison of representative liquid electrolyte systems for room-temperature Na–S batteries^a^

Electrolyte class	Electrolyte composition	Cathode	Ionic conductivity	The S content; S loading	Electrolyte/sulfur (*E*/*S*) ratio	Potential window; initial capacity; retention; long-term cycling	CE: first cycle; subsequent	Note	Ref.
Ether electrolyte	1 M NaCF_3_SO_3_ in TEGDME	S/C composite	n.r.	45%; 0.31 mg cm^−2^	6.25 µL mg^−1^ (cathode side), 12.5 µL mg^−1^ (total cell)	1–3 V *vs.* (Na/Na^+^); 1070 mAh g^−1^ of sulfur at 1/128C; A 73% retention over 37 cycles at RT.	∼84.6%; nearly 100%	Molar concentration of sulfur in the cell relative to the solubility limit of each specific polysulfide species	48
Ether electrolyte	1 M NaClO_4_ in TEGDME	SPAN	n.r.	∼46.3%; n.r.	n.r.	0.7–2.8 V *vs.* (Na/Na^+^); ∼1420 mAh g^−1^; ∼800 mAh g^−1^ over 5 cycles, with a 56.3% retention; 10 cycles	n.r.	Short-chain sulfur confinement makes carbonate chemistry viable by avoiding soluble long-chain NaPS pathways	56
Ether electrolyte with carbonate additives	1 M NaClO_4_ in TEGDME with PC					0.7–2.8 V *vs.* (Na/Na^+^); ∼720 mAh g^−1^; ∼390 mAh g^−1^ over 2 cycles, with a 41.7% retention; 10 cycles	∼58.3%; n.r.		
	1 M NaClO_4_ in TEGDME with DMC					0.7–2.8 V *vs.* (Na/Na^+^); ∼1200 mAh g^−1^; ∼300 mAh g^−1^ over 2 cycles, with a 32.5% retention; 10 cycles	∼36.7%; n.r.		
Ether electrolyte	1 M NaClO_4_ with 10% FEC in DOL : DME (1 : 1)	S@sPAN (active component 42.06 wt%, theoretical capacity −704 mAh g^−1^)	n.r.	42.02%; n.r.	n.r.	0.7–2.8 V *vs.* (Na/Na^+^); 1035 mAh g^−1^ at 0.1 A g^−1^; degradation 0.064%/cycle; 470 mAh g^−1^ at 1.0 A g^−1^ over 500 cycles	∼65–70%; ∼99–100%	Solvent effects depend on strongly sulfur chemistry and cathode architecture. Tellurium acts as a eutectic accelerator to increase electron conductivity and accelerate the diffusion of sodium ions	57
	1 M NaClO_4_ with 10% FEC in DOL : DME (1 : 1)	Te_0.04_S_0.96_@pPAN (active component 47.77 wt%, theoretical capacity −714 mAh g^−1^)	n.r.	40.97%; n.r.	n.r.	0.7–2.8 V *vs.* (Na/Na^+^); 1111 mAh g^−1^ at 0.1 A g^−1^; 601 mAh g^−1^ at 6 A g^−1^; degradation 0.031%/cycle; 894 mAh g^−1^ at 1.0 A g^−1^ over 500 cycles	∼66%; ∼100%		
Carbonate electrolyte	1 M NaClO_4_ in a mixture of EC and DMC (1 : 1, v/v) with 10% FEC	S@sPAN (active component 42.06 wt%, theoretical capacity −704 mAh g^−1^)	n.r.	42.02%; n.r.	n.r.	0.7–2.8 V *vs.* (Na/Na^+^); 234 at 6 A g^−1^; degradation 0.09%/cycle; 736 mAh g^−1^ at 0.5 A g^−1^ over 300 cycles	∼65–66%; ∼99–100%		
	1 M NaClO_4_ in a mixture of EC and DMC (1 : 1, v/v) with 10% FEC)	Te_0.04_S_0.96_@pPAN (active component 47.77 wt%, theoretical capacity −714 mAh g^−1^)	n.r.	40.97%; n.r.	n.r.	0.7–2.8 V *vs.* (Na/Na^+^); 1816 mAh g^−1^ at 0.1 A g^−1^, 629 mAh g^−1^ at 6 A g^−1^; degradation 0.015%/cycle; 970 mAh g^−1^ at 0.5 A g^−1^ over 600 cycles	∼68%; ∼100%		
Carbonate electrolytes	2 M NaTFSI in PC : FEC (1 : 1) + 10 mM InI_3_	S@MPCF	1.95 × 10^−3^ S cm^−1^, 25 °C	n.r.; ∼0.36 mg cm^−2^ (standard), from ∼0.35 to ∼4.64 mg cm^−2^ (highest)	∼50 µL mg^−1^	0.8–3 V *vs.* (Na/Na^+^); 1170 mAh g^−1^ at 0.1C; 77% over 200 cycles; 581 mAh g^−1^ over 500 cycles	79.1% (0.1C), 71.9% (0.5C), 61.6% (1C); 98.5–100.4%	Concentrated carbonate/FEC suppresses NaPS dissolution; InI_3_ simultaneously assists Na protection and sulfur conversion	58
	1 M Na[B(hfip)_4_] in EC : DMC (1 : 1) + 13 wt% FEC	Sulfurated PAN (SPAN), sulfur content 38.18%wt.±0.17wt%	6.135 · 10^−3^ S cm^−1^, RT	n.r.; ∼0.6 mg S/electrode	∼200 µL mg^−1^	0.2–3 V *vs.* (Na/Na^+^); 2441 mAh g^−1^ at 0.2C; 78.8%; 1072 mAh g^−1^ over 1000 cycles at 3C	High/stable: lower efficiency at the first cycle; 100%	Weakly coordinating anion gives high conductivity and enables SPAN cycling in carbonate electrolyte	59
	1 M NaTFSI in FDMA and MTFE (antisolvent) in a 1 : 1 ratio with 1 wt%	S@C	4.3 × 10^−3^ S cm^−1^, 25 °C	60%	n.r.	0.5–2.8 V *vs.* (Na/Na^+^); 1114 mAh g^−1^ at 0.1C; 65% retention at 0.1C over 200 cycles; 491 mAh g^−1^ at 0.3C, 419 mAh g^−1^ at 0.5C, 369 mAh g^−1^ at 1.0C over 300 cycles	66.3; 99.5%	Fluorinated solvent/antisolvent combination suppresses polysulfide dissolution and stabilizes both electrode interfaces	54
Organic carbonate electrolyte	1 M NaTFSI in pure FEC	Molten sulfur inside biomass microporous carbon (S@MRP composite)	n.r.	∼40 wt%; 0.65–1.2 mg cm^−2^	n.r.	0.1–3.0 V *vs.* (Na/Na^+^); ∼960–970 mAh g^−1^ at 0.1C; degradation 0.12% per cycle; 807 mAh g^−1^ at 0.1C over 200 cycles	n.r.; >99.5%	EC as the sole solvent triggers a unique two-step cathode-electrolyte interphase (CEI) formation to enable an efficient solid–solid conversion process of S	28
High-concentration electrolyte	2 M NaFSI in DMP/TFTFE (vol. ratio 5 : 3)	SPAN	∼0.15–0.16 mS cm^−1^, 20 °C	46%; n.r.	13.5 µL mg^−1^	0.8–2.8 V *vs.* (Na/Na^+^); 530 mAh g^−1^ at 0.5C; degradation 0.077% per cycle; 530 mAh g^−1^ at 0.5C over 600 cycles	n.r.; ∼100% under both high (45 °C) and low (10 °C) temperatures	The asymmetric DMP inner solvent and low-solvating TFTFE outer diluent form robust, highly protective CEI and SEI layers, suppressing the NaPS shuttle effect and Na dendrite growth	60
Localized high-concentration electrolyte	DME: NaFSI: TTE in a 1 : 1.2 : 1 molar ratio	Ketjenblack (KB) carbon as the S host	1.98 × 10^−3^ S cm^−1^, n.r.	n.r.; ∼3.0 mg cm^−2^ (for pouch cells)	4.5 µL mg^−1^	0.8–2.8 V *vs.* (Na/Na^+^); 922 mAh g^−1^ at 0.1C (coin cell) and 1028 mAh g^−1^ at C/20 (pouch cell); degradation 0.10%/cycle; high over 300 cycles	∼5% capacity goes to forming a solid-electrolyte interphase (SEI) on the cathode; 100% over 300 cycles	Altered solvation creates inorganic-rich interfaces, suppresses NaPS shuttle, and enables dendrite-free Na cycling	61
	6 M NaFSI in DME diluted with BTFE, 1 : 1.5 molar ratio	SPAN	n.r.	46.21 wt%; 3.0 mg cm^−2^ (coin cell), 2.0 mg cm^−2^ (pouch cell)	n.r.	0.5–3.0 V *vs.* (Na/Na^+^); 1365 mAh g^−1^ (coin cell) and 1100 mAh g^−1^ (pouch cell); 94.5% retention over 300 cycles (coin cell) and 80.7% retention over 300 cycles (pouch cell)	n.r.	Highly aggregated Na^+^–FSI^−^ solvation and minimal free DME stabilize Na and suppress soluble NaPS formation	62

a n.r. is for “not reported”.

In carbonate-based electrolyte systems, sodium polysulfides are attacked by the solvent *via* mild nucleophilic addition or substitution reactions, leading to the formation of electrochemically passivated layers at the cathode-electrolyte interface (CEI).^63^ This, in turn, results in a “quasi-solid-state” chemical reaction with reduced NaPS formation and increased cycling stability. To date, FEC can first form, *in situ*, a unique “chocolate-cookie” shaped CEI through its rapid nucleophilic reaction with NaPSs, which can be further enhanced by FEC decomposition at the lower voltage of the S@MRP cathode in high-performance RT Na–S batteries (Table 2).^28^ In this study, FEC and TFSI decomposition can form an F-rich solid-electrolyte interface (SEI) on the Na metal anode, improving interface compatibility and achieving remarkable cycle performance: 456 mAh g^−1^ after 1000 cycles at 0.5C with nearly 100% coulombic efficiency. Although these electrolytes tend to minimise polysulfide dissolution because of their lower solvating capacity for sulfur compounds, they also have disadvantages.^64,65^ CEI formation in carbonate-based electrolytes might be uncontrollable, leading to deactivation of the sulfur cathode and a drop in capacity.^63^ Various strategies, such as polymer crosslinking, copolymerisation, and additives (nanofillers and plasticisers), have been employed and are still under development to address this issue.^66^ To address this issue, the ionic liquid (IL), [P14][OTf], was added to the electrolyte as a control system.^67^ In the control electrolyte +IL system, when the battery is charged with constant current, the diffusion flux of the active material at the electrode surface remains constant, and only the active material within the diffusion distance (a model-based effective transport/reaction length) can be oxidised on the electrode surface under a given concentration difference. The increased Na_2_S/Na_2_S_2_ ratio in the control electrolyte +IL system increases the diffusion distance, allowing reuse of “dead Na_2_S/Na_2_S_2_” and dramatically enhancing sulfur utilisation as an active material.^67^ Consequently, this has led to a threefold increase in sulfide solubility and recovery of dead Na_2_S, with a capacity of 351 mAh g^−1^ over 800 cycles, and only a 0.046% capacity loss per cycle. These carbonate co-solvents and additives are incorporated into traditional ether systems, often alongside a sulfurised PAN cathode host (Table 2). SPAN cathodes alter the underlying reaction pathway by confining short-chain sulfur species within the conductive polymer network.^57^ When paired with tellurium-doped hosts (Te_0.04_S_0.96_@pPAN) and fluorinated carbonate additives (FEC), the system exhibits profound performance. Tellurium (Te) acts as an electrocatalytic accelerator that enhances both electronic conductivity and sodium-ion transport, enabling an initial capacity of 1816 mAh g^−1^ at 0.1 A g^−1^ and maintaining a stable capacity of 970 mAh g^−1^ at 0.5 A g^−1^ over 600 cycles, with degradation of only 0.015% per cycle. Alternatively, fluorinated-solvent strategies offer benefits, including a stable CEI, reduced flammability, and improved compatibility with sodium metal, as fluorination weakens solvent polarity and suppresses polysulfide dissolution.^68^ For this reason, researchers have introduced fluorinated solvents such as DME-TTE, 2,2,2-trifluoro-*N*,*N*-dimethylacetamide (FDMA), and 1,1,2,2-tetrafluoroethyl methyl ether (MTFE). In ref. 54, the fluorinated solvent (FDMA)/antisolvent (MTFE) combination, with ionic conductivity of ∼4.3 mS cm^−1^ at 20 °C, suppresses polysulfide dissolution and stabilises both electrode interfaces, resulting in an initial capacity of 1114 mAh g^−1^ at 0.1C with 65% retention at 0.1C over 200 cycles (Table 2). MTFE most likely contributes by reducing the number of solvent donors and weakening the solvation shell, thereby limiting polysulfide dissolution. To further improve interfacial stability and ionic conductivity, localised saturated electrolytes, such as 2-methyltetrahydrofuran (MeTHF)-TTE, have emerged. MeTHF functions as a weakly Na^+^-solvating solvent, promoting rapid Na^+^ desolvation kinetics due to its Gibbs binding energy.^69^ DFT simulations show that the binding energy between the Na^+^ cation and the solvent (Δ*G*_binding_) decreases in the order of DME (−45.1 kcal mol^−1^) > MeTHF (−32.58 kcal mol^−1^) > THF (−31.59 kcal mol^−1^) > THP (−30.47 kcal mol^−1^).^70^ DME shows solvating capability because of its bidentate nature and higher polarity.

A comparative evaluation of carbonate- and ether-based electrolytes reveals a classic balance: ethers struggle with the sulfur cathode because of polysulfide dissolution, while carbonates impair the metal anode because of poor interphase stability. However, as reported by ref. 71 tailoring the thermal annealing process of carbon–sulfur composites at 155 °C (155S) and 300 °C (300S) offers a means to alter these electrolyte interactions. The 155S electrode, containing up to 72% sulfur, delivered a reversible capacity of 483 mAh g^−1^ in an ether electrolyte at a current density of 0.1 A g^−1^, compared with 279 mAh g^−1^ in the carbonate ester system. This disparity highlights how spatial sulfur distribution dictates the phase-transformation pathway: surface-located sulfur (155S) enables solid–liquid conversion in an ether electrolyte, whereas in a carbonate electrolyte, it leads to parasitic reactions. Conversely, for pore-confined sulfur (300S), solid–liquid conversion is confined in an ether-based system, whereas a carbonate-based electrolyte leads to less efficient solid–solid conversion.

Beyond these approaches, advanced electrolyte design focuses on manipulating solvation structure *via* weakly coordinating anions and (localised) high-concentration electrolytes, and more recently localised saturated electrolyte (LSE) strategies.^24^ The main focus has shifted to high-concentration electrolyte (HCE) systems to improve cycling stability and interfacial compatibility. Highly concentrated carbonate-based electrolytes are common here. In ref. 60 (Table 2), a novel high-concentration Na–S electrolyte system (NaFSI-DMP/TFTFE), comprising two solvents-1,2-dimethoxypropane (DMP) and 1,1,2,2-tetrafluoroethyl-2,2,2-trifluoroethyl ether (TFTFE) as the inner solvent and outer diluent, respectively-is used to inhibit the shuttle effect. Remarkably, it achieves a 530 mAh g^−1^ discharge capacity at 0.5C over 600 cycles, with a capacity fade of only 0.077% per cycle. Because of its methyl groups, DMP plays a distinct role in regulating polysulfide dissolution and transport by weakening the ion–dipole interaction between Na^+^ and oxygen atoms, thus promoting initial NaPS dissolution compared to DME. Its steric hindrance limits polysulfide solvation, reducing their equilibrium concentration and long-range diffusion in the electrolyte. The external solvent, TFTFE, ensures extremely low NaPS solubility, suppresses polysulfide dissolution and the shuttling effect, and further localises the active species near the cathode. Compared to DME, DMP's electron-donating methyl groups increase oxygen electron density, resulting in higher binding energy (−45.1 kcal mol^−1^) and a lower LUMO (−0.67 eV).^60^ Furthermore, adding TFTFE (HOMO value: −8.82 eV) enhances oxidative stability and restricts NaPS dissolution. This unique structure forces FSI^−^ into the Na^+^ solvation sheath, forming an inorganic-rich SEI and a uniform CEI. These protective interphases suppress sodium dendrites and electrode side reactions, drastically improving cell stability. However, despite favorable solvation characteristics, HCEs can exhibit high viscosity and pronounced ion association, which reduce particle diffusion coefficients and overall ionic conductivity.^24,54^ In addition, overall ionic conductivity reflects not only Na^+^ transport, but also all transport properties collectively, including the Na^+^ transference number and correlated ion dynamics.^72^ Thus, under sustained current, insufficient Na^+^ transport can lead to concentration polarisation. Another reason limiting the practical application of highly concentrated electrolytes is their high cost.^24,54^

Transitioning from high-concentration electrolytes (HCEs) to localized high-concentration electrolytes (LHCEs) aims to reduce viscosity and costs while maintaining favourable electrochemical performance.^24^ A key feature of LHCEs is their ability to partially decouple macroscopic transport properties from the local coordination environment of Na^+^ ions.^73^ In conventional HCEs, extensive cation–anion association and a lack of free solvent molecules lead to contact ion pairs and larger aggregates; however, high salt content also elevates viscosity, reduces wettability and restricts mass transport. In contrast, LHCEs overcome these limitations by incorporating a low-viscosity diluent (weakly coordinating or essentially non-solvating) that is miscible with the base HCE yet has a significantly lower affinity for Na^+^ than the coordinating solvent.^74^ Thus, the term “localised concentration” refers to the spatial localisation of anion-rich, salt-enriched coordination domains within a dilute, fluid medium on a macroscopic scale rather than bulk salt concentration.^73^ This configuration balances interface stability and ion mobility, facilitating sustained cycling stability, mitigating dendrite formation, enhancing oxidative durability, and improving overall electrochemical performance under challenging operational conditions. Such electrolyte systems mitigate the core physical and chemical constraints on ion-transport dynamics, charge-carrier mobility, and stability at the electrode–electrolyte interface in sodium batteries.^73^ To date,^29^ reports that sodium bis(trifluoromethanesulfonyl)imide, sulfolane solvent, and 1,1,2,2-tetrafluoroethyl-2,2,3,3-tetrafluoropropyl ether diluent in a 1 : 2 : 2 molar ratio, paired with a pre-sodium activated carbon cloth (NaACC) anode, support a stable and safe room-temperature Na–S battery. The RT Na–S battery shows a high initial capacity of 1220.8 mAh g^−1^, favourable rate capability, and stable cycling performance, with an average coulombic efficiency of 99.1%. After 100 cycles, the capacity remains at 704.5 mAh g^−1^. This strong performance may be due to the electrolyte system greatly reducing the polysulfide shuttle effect, enabling the formation of a stable anion-derived NaF-rich cathode electrolyte interphase, and achieving a solid–solid conversion mechanism of solid S_8_ to solid Na_2_S_*n*_ (1 ≤ *n*≤ 3). However, even with the latest LHCE, uncontrolled Na electrodeposition occurs at an overpotential >1.2 V at low temperature, causing cell failure within hours.^73^ To address this, IL-tailored LHCE is used in durable sodium electrodeposition and shuttle-free 0.5 Ah Na–S pouch cells that operate at −20 °C.^75^ This electrolyte system promotes anion and solvent exchange within the solvation shell at reduced temperatures, involving Na^+^ and organic cations. In Na–S batteries employing SPAN cathodes, LHCE-Toluene has been shown to mitigate the shuttle phenomenon, promote sulfur conversion from solid-to-solid forms, minimise active material loss, and reduce erosion of the sodium-metal anode.^76^ As a result, Na-SPAN cells exhibit significantly enhanced cyclability, with 85% capacity retention after 200 cycles at a C/2 rate and 64% after 300 cycles at a 1C rate, along with a low capacity-fade rate of 0.12% per cycle. In a localised system composed of DME, NaFSI, and TTE paired with Ketjenblack carbon–sulfur host, ionic conductivity reaches ∼1.98 mS cm^−1^ (Table 2).^65^ In a pouch cell configuration with S loading of ∼3.0 mg cm^−2^, it delivered 1028 mAh g^−1^ at C/20, with capacity degradation of 0.10% per cycle over 300 cycles. Similarly, in ref. 62, a 6 M NaFSI in DME system with BTFE as a diluent and a SPAN cathode achieves 1365 mAh g^−1^ and 1100 mAh g^−1^ in coin cell and pouch cell configurations, respectively, over 300 cycles. Advanced LHCEs enhance sulfur redox and conversion kinetics by modulating Na^+^-solvent interactions, reducing free solvent molecules, and fostering anion-dominated interphases. This tailored environment facilitates sulfur-species dissolution and conversion at the cathode, while limited free-solvent activity and strong ion–solvent associations restrict polysulfide solubility and diffusion toward the Na-metal anode. Consequently, this setup ensures efficient cathode reactions, minimises polysulfide crossover, and supports dendrite-free cycling under practical conditions. However, LHCE design needs to regulate electrolyte composition and structure by adjusting salt-to-solvent ratios to create localised, anion-rich environments, selecting functional salts to influence interphase morphology and conductivity, and adding inert diluents to reduce viscosity while preserving ion transport.^62,65^ Incorporating multifunctional additives and co-solvents also improves interfacial stability and suppresses parasitic reactions. Developing composite co-solvent systems enables precise control over viscosity and solvation, with computational methods such as molecular dynamics and density functional theory aiding the optimisation of salt and solvent selection. These combined strategies, supported by characterisation and electrochemical testing, enable the identification of optimal salt-to-solvent ratios for LHCEs.

### 4.2 Solid-state electrolytes

Conventional high-temperature Na–S batteries use β-Al_2_O_3_ as a Na^+^-conducting ceramic separator and operate with molten electrodes, typically around 300–350 °C. In an all-solid-state Na–S battery, the electrolyte must simultaneously provide Na^+^ conductivity, block electron transport and macroscopic polysulfide migration, remain sufficiently stable against highly reducing Na-based anodes, establish intimate contact with insulating sulfur/Na_2_S, and accommodate very large conversion-induced volume changes.^77^ However, once the ionic conductivity of a solid electrolyte reaches ≈10^−3^ S cm^−1^, overall performance is more frequently constrained by cathode-scale transport, interfacial reactions, and chemo-mechanical contact than by bulk electrolyte resistance.^78^ Consequently, increasing bulk electrolyte conductivity cannot, by itself, guarantee full sulfur utilisation if sulfur conversion is limited by solid-state diffusion, interfacial contact, or reaction kinetics. To overcome these challenges, different solid-state electrolyte platforms are emerging for RT Na–S batteries. One is sulfide solid electrolytes, such as Na_3_PS_4_ and Na_3_SbS_4_, which combine room-temperature Na^+^ conductivity with mechanical stability greater than that of dense oxides.^79^ This allows particles to deform under pressure and generate favourable solid–solid contact areas; however, their apparent compatibility with sulfur must be controlled because sulfides can oxidise or reduce at both electrodes. Na_3_SbS_4_ is particularly attractive because Na vacancies and its three-dimensional Na-ion transport network make its conductivity highly responsive to substitutional chemistry. To date, simultaneous cation/anion modification of Na_3_SbS_4_, codoping with W and Cl, altered the local environment and Na-ion migration landscape, producing an RT conductivity of ≈6.4 mS cm^−1^ while broadening electrochemical tolerance.^78^ Most importantly, this study demonstrates chemically programmable sulfide electrolytes in which cation substitution leads to framework distortion and favourable Na-vacancy energetics, while anion substitution drives polarizability, local bonding, and electrochemical stability. In ref. 80, Ca^2+^ substitution into the Na_3_SbS_4_ structure generates Na vacancies and facilitates vacancy-mediated Na^+^ migration. With an optimised structure, Na_2.9_Ca_0.05_SbS_4_ achieves 0.51 mS cm^−1^ and provides improved electrode compatibility and strong capacity retention. Furthermore, according to ref. 81, electrolyte decomposition might not be purely detrimental for the battery. As reported, the Na_3_SbS_4_ electrolyte undergoes redox transformation at the sulfur electrode. However, if decomposition is spatially controlled and produces mechanically coherent interphases that are electronically blocking and Na^+^-conducting, it is useful. Such an electrolyte achieves ≈ 1976 mAh g^−1^ specific capacity, dropping to ≈1295 mAh g^−1^ over 140 cycles at 0.127 mA cm^−2^.

Among solid electrolytes for Na–S batteries, Na_3_PS_4_ has become a model electrolyte for resolving the reaction mechanism of sulfur under solid-state conditions. According to ref. 32, the intrinsic Na–S reaction is complex and, at extremely low rates or elevated temperatures, proceeds thermodynamically as follows [chem eqn (5)]:

5 S_8_ → Na_2_S_5_/Na_2_S_4_ → Na_2_S_2_ → Na_2_S,

While at practically more relevant temperatures and faster rates, the Na_2_S_2_ step is kinetically bypassed, producing the more familiar two-step electrochemical response. If sulfur conversion is kinetically restricted, increasing electrolyte conductivity cannot guarantee complete utilisation, as Na^+^ transport through the electrolyte is only part of the reaction resistance.^32^ This suggests that, rather than further increasing conductivity, it would be more beneficial to redesign the sulfur/SSE/carbon microstructure.

Regarding the behaviour of solid electrolytes towards polysulfide intermediates, they can suppress long-range dissolution and migration of polysulfides but do not necessarily suppress their formation as solid or confined intermediates. In ref. 50, an *operando* study shows that in Na–S conversion with both Na_3_PS_4_ and the hydroborate electrolyte, the system contains different crystalline and amorphous long- and short-chain Na_2_S_*x*_ intermediates under appropriate local conditions, highlighting that the electrolyte changes Na–S chemistry and leads to a spatially confined multiphase conversion process. Here, hydroborate (Na_4_(B_10_H_10_)(B_12_H_12_)) improves capacity and cycling behaviour due to its higher Na^+^ conductivity (4.07 mS cm^−1^), delivering 500 mA g^−1^ at 30 °C. Moreover, sulfur reaction pathways depend on local mechanical stress:^50^ diffusion-limited phase transformations and localised stress determine which polysulfide phase forms by controlling contact area, local chemical potential, and phase stability. In other words, pressure strongly contributes to the thermodynamics and kinetics of sulfur conversion.

Another important property of solid-state electrolytes is chemo-mechanical compatibility. A central advantage of sulfide electrolytes over NASICON ceramics in bulk composite cathodes is mechanical deformability.^82^ Dense NASICON-type electrolyte is intrinsically brittle and produces poor conformal contact with sulfur cathodes. However, once converted into a flexible film, a ceramic-rich NASICON membrane, <200 µm thick, with a relatively moderate conductivity can outperform a more conductive but fabricated substantially thicker one in Na–S batteries.^83^ In this case, considering that repeated cycling can separate sulfur-derived particles from the SSE, break the carbon/S/SSE percolating network, create voids, generate cracks, increase local current density, and accelerate interfacial decomposition, the lack of a standard metric for the effects of elastic modulus, fracture toughness, interfacial energy of the solid electrolyte and separator thickness is an important research gap. In solid sulfide electrolytes, materials-structure and defect engineering can improve conductivity and control interface chemistry, while oxide-polymer composites, through electrolyte architectural optimisation, reduce SSE thickness and increase flexibility and contact area.

Alternatively, gel polymer electrolytes are also highly effective. In ref. 84, an *in situ*-crosslinked pentaerythritol triacrylate-based gel polymer electrolyte showed an ionic conductivity of approximately 2.33 mS cm^−1^ at 25 °C and retained more than 600 mAh g^−1^ after 2500 cycles, with an average coulombic efficiency near 99.91%. According to ref. 85, a multifunctional crosslinked polymer interface confines sulfur species and enables quasi-solid sulfur conversion, making quasi-solid sulfur chemistry a deliberate design target. The electrode retained approximately 712 mAh g^−1^ after 150 cycles, with an initial areal capacity of 5.84 mAh cm^−2^ at a sulfur loading of 3.18 mg cm^−2^. More broadly, these results point to a general design principle for quasi-solid electrolytes: optimal architectures should prioritise spatial confinement over maximum rigidity, provided local molecular mobility is preserved to support rapid sulfur conversion chemistry.

Another emerging concept is that the boundary between electrolyte and active material is becoming less distinct. In ref. 86, mechanical formation of a Na_2_S–NaI solid solution, technically representing more of an active cathode phase than the separator electrolyte, reportedly increased Na-ion conductivity by around a 5-order-of-magnitude relative to Na_2_S. Thus, recent progress in solid electrolyte design focuses on conductivity engineering *via* manipulating Na vacancies, local lattice geometry and cation/anion chemistry; interfacial reactivity, turning the electrolyte decomposition functional; and new insights into the Na–S mechanism, the role of pressure and mechanics, thin solid electrolytes, and quasi-solid chemistry as a possible compromise, with major questions remaining: optimisation of the electrolyte content and ionic conductivity, which might not be the most effective metric for all solid electrolytes.

### 4.3 High-entropy electrolytes

Under the modern concept of high-entropy electrolytes (HEE), increasing the molecular diversity of electrolyte compositions can promote favourable ion coordination and interfacial chemistry in lithium-metal batteries.^87^ Rather than optimising traditional variables (such as salt type, concentration, and solvent composition), the high-entropy strategy relies on the synergistic action of multiple chemical components—differing in chemical structure and properties—to create a statistically diverse solvation environment, where the distribution of molecular configurations is governed by ion–solvent and ion–anion interactions. In ref. 88, a single-solvent high-entropy electrolyte (based on diethylene glycol dibutyl ether solvent and NaPF_6_ salt) was used in practical high-voltage anode-free sodium pouch cells, demonstrating cyclability in a wide temperature range – from −20 °C to 60 °C. For sodium-ion batteries, integrating entropy-driven chemistry (desolvation behaviour of Na^+^, ionic conductivity, and low-temperature activity) with rational designs of both liquid and solid electrolytes is marked as a promising future research direction.^89^ Similarly, for Na–S batteries, the primary advantage of HEEs might lie in their molecular diversity-arising multifunctionality. Strong anion interactions and relatively weak cation solvation drive high configurational complexity, facilitating salt dissociation and contact ion-pair formation.^90^ This formation chemistry stabilises anion-derived electrode/electrolyte interphases and enables operation across a wide temperature range.^77^ In electrolytes with diverse Na^+^ coordination environments, SEI formation primarily involves anion decomposition. Because electrolyte instability hampers room-temperature sodium–sulfur cell operation, this configurational entropy may help regulate the probability distribution of Na^+^ coordination environments.^91^

Traditional electrolyte strategies often aim to minimise polysulfide solubility; however, completely eliminating soluble intermediates is not always optimal, as sulfur conversion is a complex multiphase reaction.^77^ Consequently, the focus shifts from absolute polysulfide suppression to targeted solvation engineering, achieved *via* integrated electrolyte and separator designs to maintain sufficient local mobility for charge transfer while preventing long-range migration. An optimal electrolyte should facilitate ion transport and interfacial conversion while preventing long-range polysulfide migration. Here, a multicomponent HEE can address two key challenges: controlling polysulfide release into the electrolyte and managing their processes after dissolution.^87,92^ Through effective Na^+^ ion coordination and regulation of viscosity or dielectric constant, such electrolyte systems can promote interface formation or polysulfide immobilisation, creating a heterogeneous solvation environment.^92^ This environment provides multiple energetically competing coordination sites for sodium ions, electrolyte anions, and polysulfide species. With the right configuration, it can enable rapid Na^+^ exchange while simultaneously hindering long-range polysulfide transport, accounting for the chemically diverse sulfur intermediates—long-chain Na_2_S_*x*_ and short-chain polysulfides (Na_2_S_2_ and Na_2_S)—that interact differently with solvents. However, despite promising prospects, high-entropy electrolyte development for sodium–sulfur batteries remains an emerging research area rather than an established technology.^93,94^ The first challenge lies in determining the configurational entropy.^95^ Electrolyte solutions are non-ideal systems characterised by strong ion–dipole, ion–ion, and intermolecular interactions; consequently, simple entropy calculations fail to capture the effective entropy associated with Na^+^ solvation. Secondly, electrolyte characterisation becomes increasingly complex as the number of components rises: Raman or IR spectroscopic bands may overlap, and standard NMR spectra can become convoluted. Therefore, molecular dynamics simulations—potentially augmented by machine learning—may be essential for determining the probability distribution of Na^+^ coordination environment. Thirdly, given the vast array of possible compositions for high-entropy electrolytes, the experimental trial-and-error approach proves inefficient.^93,94^ This has driven significant interest in Na–S high-entropy electrolytes regarding high-throughput experimentation, molecular modelling, Bayesian optimisation, and machine-learning-assisted electrolyte discovery. Finally, electrolyte performance must ultimately be demonstrated under realistic cell operating conditions. Key evaluation criteria should include high sulfur loading, a low electrolyte-to-sulfur ratio, limited sodium inventory, and practically relevant current densities. An electrolyte that sustains hundreds of cycles under excess sodium and large electrolyte volumes may prove ineffective in a commercially viable cell. Collectively, this underscores the importance of a deep understanding of reaction pathways and degradation mechanisms in Na–S systems—including electrolyte evolution—as well as the need for *in situ* investigation methods,^96^ an *operando* correlational approach,^97^ together with theoretical modelling and machine learning to obtain more accurate quantitative data and improve predictive capabilities.

## 5 Separator engineering and interlayer design in room-temperature Na–S batteries

In sodium–metal batteries (SMBs), the separator largely determines the reliability and durability of the entire electrochemical cell.^98^ Unlike conventional rechargeable systems with solid anode materials, where the separator's primary role was limited to physically separating the electrodes and retaining the electrolyte, metallic sodium gives the separator a fundamentally new role.^99,100^ Na's high reactivity, dendrite formation during plating and stripping, and significant anode volume fluctuations inevitably intensify interfacial processes.^101^ Therefore, the separator can no longer be regarded as a “passive barrier” but rather as a functional element that governs ion transport, SEI stability, and the distribution of local current densities.^102–104^ A properly selected or modified separator can address several critical challenges simultaneously.^22^ First, it ensures a uniform Na^+^ flux across the electrode–electrolyte interface, thereby reducing local overpotentials and minimising “tip effects” that promote dendrite growth.^105^ Second, it helps stabilise the SEI, which tends to rupture and reform under cycling, leading to sodium loss and reduced coulombic efficiency.^106,107^ Third, the separator must exhibit sufficient mechanical strength and thermal stability to withstand cycling-induced stresses and prevent short-circuit failure under practical conditions.^108–110^ As Wang *et al.*^22^ highlight, recent research has focused on functionalizing separators, ranging from interfacial chemistry regulation to multiscale structural engineering and the incorporation of hybrid organic–inorganic materials. These approaches not only improve electrolyte wettability and mechanical robustness but also enable more uniform ion flux distribution, suppress side reactions, and stabilise interfacial processes.^102^

The separator is especially critical in Na–S systems, where it must control dendrite growth at the sodium anode while suppressing polysulfide diffusion through the electrolyte.^22,108^ In this case, the separator serves as a dual-functional platform: it regulates Na^+^ behaviour and protects the anode while mitigating the polysulfide shuttle effect.^107^ This comprehensive role makes the separator a key element that directly determines the feasibility of safe, energy-dense, and long-lasting Na–S batteries.

From a mass-transport perspective, separator selectivity requires suppressing soluble NaPS crossover while maintaining efficient Na^+^ transport. Diffusive crossover across a membrane can be described in terms of permeability and the concentration difference across the membrane, with membrane thickness entering the Fickian description of permeation.^111^ Charge- and size-exclusion effects can further decrease the permeability of crossing species.^111^ In RT Na–S batteries, NaPS crossover can also be regulated through adsorption at functional separator or interlayer surfaces, while catalytic components can promote subsequent polysulfide conversion.^112–115^Table 3 summarises different separator and interlayer engineering strategies developed to meet these requirements.

**Table 3:** Separator and interlayer engineering strategies for RT Na–S batteries^a^

Separator/interlayer design	Functional material/modification	Main function/NaPS-control mechanism	Ionic conductivity, mS cm^−1^	Polysulfide rejection/permeability	Mechanical properties	Current rate/density	Initial capacity, mAh g^−1^	Capacity, mAh g^−1^ after (*n*) cycles	Ref.
Gradient-engineered asymmetric separator (GES)	ZIF-67/ZnF_2_ on PP	ZIF-67: Physical confinement, Co–S adsorption, catalytic NaPS conversion; ZnF_2_: Homogeneous Na^+^ flux, NaF-rich SEI	0.41	Receiving compartment nearly colorless after 24 h	Tensile strength: 2.8 kN m^−1^	0.5 C	1028.6	778.7(400)	116
Biomimetic ion-selective COF membrane	HB/CNT@COF	Size sieving, electrostatic NaPS repulsion, polysulfide conversion	n.r.	Almost no Na_2_S_6_ permeation after 24 h	n.r.	4C	n.r.	733.4(400)	117
Giant molybdenum cluster-modified GF separator	{Mo_154_} clusters on glass fiber (GF/Mo_154_)	Lewis acid–base NaPS adsorption, bidirectional electrocatalytic conversion	0.24	Almost no Na_2_S_6_ permeation after 192 h	n.r.	1C	1457.1	1037.6(4000)	118
Sandwich-structured separator	PI-FPU/Celgard/PI-FPU	Physical confinement, chemical anchoring of NaPSs	0.54	Almost no Na_2_S_6_ permeation after 48 h	n.r.	3A g^−1^	n.r.	555(1500)	112
Electrospun nanofiber separator	PI–PEO	Dendrite suppression, uniform Na^+^ transport	1.48	n.r.	Elongation at break: 126.2%; puncture force: 3.56 N; puncture displacement: 6.73 mm	3A g^−1^	n.r.	450(2000)	113
Catalytic Fe_3_Se_4_@NPCN-coated GF separator	Fe_3_Se_4_ nanoparticles anchored on N-doped porous carbon nanosheets	Physical confinement, Fe-site chemisorption, catalytic NaPS conversion	n.r.	Almost no Na_2_S_6_ permeation after 24 h	n.r.	1A g^−1^	n.r.	340(500)	114
Hierarchical hybrid MXene interlayer	Ti_3_C_2_T_*x*_/Mo_2_Ti_2_C_3_T_*x*_ on GF	Physical blocking, chemical adsorption, catalytic NaPS conversion, Na^+^ flux regulation	n.r.	n.r.	n.r.	1C	562	357(500)	115

a n.r. is for “not reported”.

As shown in Table 3, the reported separator architectures emphasize different functions. In adsorption- and catalysis-based systems, soluble NaPSs are retained by polar or chemically active sites, while their conversion is promoted by catalytic components, as demonstrated for the ZIF-67/ZnF_2_ and Mo_154_-based separators.^116,118^ In ion-selective designs, molecular sieving and electrostatic repulsion restrict polysulfide migration, while the membrane maintains Na^+^ transport.^117^ In polymer-based sandwich and electrospun separators, NaPS confinement or Na^+^ transport regulation is combined with interfacial and mechanical stabilization.^112,113^ A hierarchical MXene interlayer provides a different combination of functions, integrating physical blocking, chemical adsorption, catalytic NaPS conversion, and Na^+^ flux regulation within the same architecture.^115^

Table 3 also provides quantitative data for several of these separator designs, although the reported parameters vary considerably among studies. Ionic conductivities of 0.41 and 0.54 mS cm^−1^ were reported for the gradient-engineered and sandwich-structured separators, respectively,^112,116^ while the electrospun PI–PEO separator showed a value of 1.48 mS cm^−1^.^113^ Na_2_S_6_ permeation was limited after 24 h for the biomimetic COF membrane^117^ and after 48 h for the sandwich-structured separator,^112^ whereas negligible permeation was reported after 192 h for the Mo_154_-modified GF separator.^118^ Mechanical properties are reported less consistently: a tensile strength of 2.8 kN m^−1^ was given for the gradient-engineered separator,^116^ while an elongation at break of 126.2% and a puncture displacement of 6.73 mm were reported for the PI–PEO separator.^113^ Because these values were obtained under different experimental conditions, they are more appropriately considered as reported characteristics of individual separator designs rather than as a direct ranking of their performance.

Interlayer engineering has also enabled functions beyond polysulfide blocking. In a hierarchical Ti_3_C_2_T_*x*_/Mo_2_Ti_2_C_3_T_*x*_ MXene interlayer, physical blocking and chemical adsorption were combined with catalytic NaPS conversion and Na^+^ flux regulation.^115^ In another approach, an NZSP interlayer provided polysulfide anchoring and mechanical confinement to mitigate chemomechanical fatigue of the sulfur cathode.^119^ Capacities of 830.56 mAh g^−1^ after 500 cycles at 2 A g^−1^ and 489.81 mAh g^−1^ after 2000 cycles at 5 A g^−1^ were retained with the NZSP-modified cathode.^119^ These studies show that an interlayer's function is not limited to NaPS crossover suppression but can also include regulation of conversion kinetics, ion transport, and structural stability.

The comparison in Table 3 also shows that the reported advantages span different separator properties rather than a single parameter. Different separator designs have demonstrated higher ionic conductivity, prolonged suppression of NaPS permeation, mechanical flexibility, and long-term cycling stability under different testing conditions.^112–115^ Direct numerical comparison should therefore be made with some caution, since the reported values were obtained using different permeation protocols, current rates, cathode compositions, and cell configurations.

### 5.1 Janus separator design for Na–S batteries: structure–function relationship

Among emerging separator engineering strategies, Janus-type architectures have attracted increasing attention because they introduce spatially separated functionalities within a single separator.^120^ Unlike conventional designs, Janus separators enable directional control by assigning distinct roles to each side. Typically, the anode-facing side is modified with ion-conductive or polymer-grafted layers to regulate Na^+^ flux and stabilise sodium deposition,^121^ while the cathode-facing side incorporates polar or catalytic materials to adsorb and convert polysulfides.^122^ A representative Janus separator design reported in the literature combines an ion-selective polymer-grafted layer with catalytic materials, enabling simultaneous regulation of Na^+^ transport and suppression of polysulfide migration.^50^ Such Janus architecture highlights the shift from passive barriers to active functional platforms.^120^ This strategy represents a promising direction in separator engineering to improve ion transport, suppress polysulfide shuttling, and enhance the overall electrochemical performance of Na–S batteries.^123^

### 5.2 Ion-selective separator design

Beyond asymmetric (Janus) architectures, ion-selective separator design has emerged as an effective strategy to regulate ion transport at the molecular level.^124^Fig. 6 illustrates a representative example in which a MIL-121/Na-based separator is designed to form sodium-ion-selective channels. As shown, negatively charged functional groups electrostatically repel polysulfide species. At the same time, the porous structure provides physical screening, effectively blocking the diffusion of larger polysulfide molecules.

**Fig. 6:**
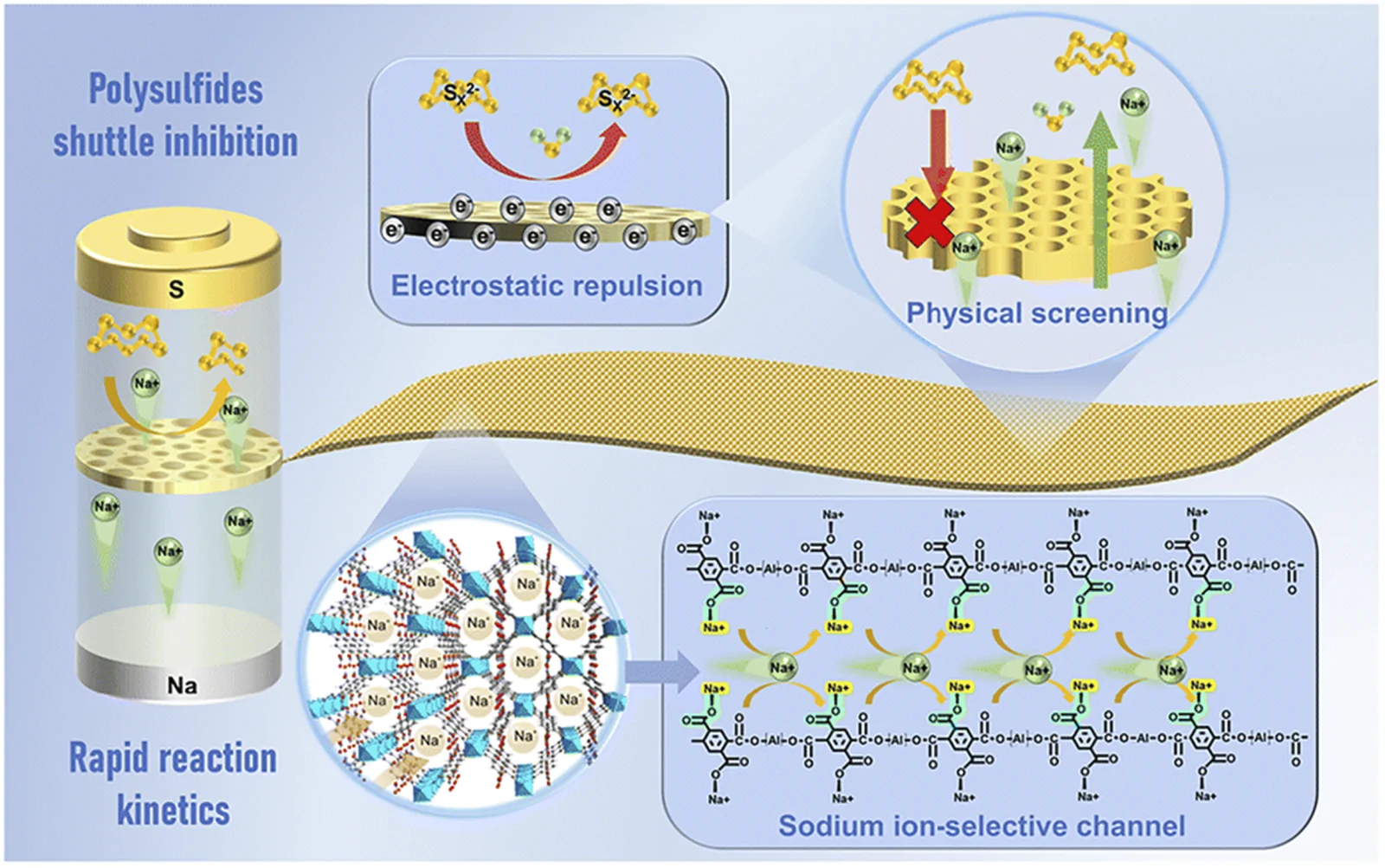
Schematic of the design of the MIL-121/Na sodium ion channel. Reproduced from ref. 124 with permission from the Royal Society of Chemistry, copyright © 2026.

The interconnected ion pathways simultaneously facilitate Na^+^ transport, as reflected by an ionic conductivity of 2.27 × 10^−4^ S cm^−1^ and a Na^+^ transference number of 0.74.^124^ In an H-cell permeation test, no significant polysulfide migration through the MIL-121/Na separator was observed after 48 h, whereas polysulfides had nearly completely crossed the GF separator within approximately 3 h.^124^ This dual mechanism, combining electrostatic exclusion and size-selective transport, plays a key role in suppressing the shuttle effect and stabilising electrochemical processes.^124^ At the same time, the interconnected ion pathways enable fast Na^+^ transport, improving ionic conductivity and reaction kinetics. This dual mechanism, combining electrostatic exclusion and size-selective transport, plays a key role in suppressing the shuttle effect and stabilising electrochemical processes.^124^ Such separator systems can be regarded as bio-inspired designs that mimic selective ion transport in biological membranes.^125^ This concept highlights an emerging direction in separator engineering, in which precise control of ion transport is achieved through rational structural and chemical design.^126^ Overall, ion-selective separator engineering offers a promising pathway to improve the stability and performance of Na–S batteries, complementing advanced approaches such as Janus architectures.^120,124^

## 6 Research prototypes of Na–S battery systems

In Na–S batteries, the sulfur cathode is considered both a source of high theoretical capacity and the main obstacle to electrochemical stability. At the anode, separator, electrolyte, and cell-configuration levels, researchers have proposed a wide range of engineering approaches to stabilise Na–S systems and mitigate the primary degradation mechanisms. However, researchers can evaluate the effectiveness of these approaches only in fully assembled cells, not in individual components. In this context, this work focuses on Na–S battery prototypes that integrate optimised Na metal anodes, sulfur-based cathodes, customised electrolytes, and modified separators into a single architecture. In the following section, we systematically analyse Na–S cell prototypes and summarise their configurations and electrochemical performance, including specific capacity, cycling stability, and energy efficiency. This section systematically discusses Na–S cell prototypes and summarises their configurations and electrochemical performance, including specific capacity, cycling stability, and energy efficiency, in Table 4.

**Table 4:** Research prototypes of Na–S cells with Na metal anodes (2015–2026)

Positive electrode material	Sulfur content, wt%	Sulfur loading, mg cm^−2^	Areal capacity, mAh cm^−2^	*E*/*S* ratio, µL mg^−1^	Electrolyte	Current rate/density	Initial capacity, mAh g^−1^	Capacity, mAh g^−1^ after (*n*) cycles	Ref.
SPAN web	41	n.r.	n.r.	n.r.	1 M NaPF_6_ in EC/DMC (1 : 1)	0.1C	756	648(200)	127
S@iMCHS	60	n.r.	n.r.	n.r.	1 M NaClO_4_ in PC/EC (1 : 1) with 5% FEC	100 mA g^−1^	1215	292 (200)	128
Na_2_S/MWCNT	n.r.	n.r.	n.r.	n.r.	1.5 M NaClO_4_ and 0.3 M NaNO_3_ in TEGDME	C/10	660	560 (50)	129
S@Con/HC	47	n.r.	n.r.	n.r.	1 M NaClO_4_ in PC/EC with 5wt% FEC	100 mA g^−1^	1081	508(600)	57
S@MPCF	61.09	n.r.	n.r.	n.r.	2 M NaTFSI in PC/FEC with InI_3_	0.1C	1170	927(200)	130
(C/S/BaTiO_3_)@TiO_2_ nanofiber cathode	60	n.r.	n.r.	n.r.	1 M NaClO_4_ in EC/DEC (1 : 1)	1 A g^−1^	952	524.8(1400)	131
SC-BDSA	40.1	3	n.r.	n.r.	1 M NaClO_4_ in EC/PC (1 : 1)	250 mA g^−1^	1057	784(200)	132
Se_0.08_S_0.92_@pPAN	36.88	n.r.	n.r.	n.r.	1 M NaTFSI in TEGDME with 5% FEC	0.4 A g^−1^	1185	770(500)	133
SPAN	43	1.2–1.5	n.r.	100	2M NaTFSI/TMP in FEC	1C	1025	788(300)	134
S-PAN cathode with PAA binder	41	2.5	n.r.	n.r.	1 M NaClO_4_ in EC/PC/FEC/SnCl_2_	0.5C	1195	1000(1000)	135
Co@NPCNFs/S	38	1	n.r.	n.r.	1 M NaClO_4_ in EC/DEC (1 : 1)	1C	906 (0.1C)	411(800)	136
Covalent sulfur–carbon composite (covalent-SC)	36.9	n.r.	n.r.	n.r.	1 M NaClO_4_ in EC/DEC (1 : 1) with 5% FEC	0.8C	1069	888(600)	137
N-MXene@MWCNT-MP/S (N-doped Ti_3_MXene microspheres)	73.8	5.5	n.r.	n.r.	2 M NaTFSI in PC/EC (1 : 1) with 5 wt% FEC	2C	980 (0.5C)	450(1000)	58
SPAN	38.18	n.r.	n.r.	n.r.	1 M Na[B(hfip)_4_] in EC/DMC with 13 wt% FEC	3C	1360	1072(1000)	59
S@Amorphous SiO_2_	73.5	n.r.	n.r.	n.r.	1 M NaClO_4_ in EC/PC with 5% FEC	1 A g^−1^	1106	955.8(1460)	138
S/CoS_2_-CoSe_2_@CNF_s_	45	n.r.	n.r.	n.r.	2 M NaTFSI in PC/FEC	0.1 A g^−1^	1295	869(100)	139
WBMC@S	40	1	n.r.	n.r.	1 M NaClO_4_ in EC/PC (1 : 1) with 5% FEC	0.2C	1413	822(100)	140
S@Co_1_–CoS_2_/NC	54	n.r.	n.r.	n.r.	1 M NaClO_4_ in EC/PC (1 : 1) with 5% FEC	0.2 A g^−1^	941	642(150)	141
S/ELSC	40	1	n.r.	n.r.	1 M NaClO_4_ in EC/PC with 5% FEC	1C	1395 (0.2C)	518(500)	142
CNF/CoFe_2_O_4_/S	60.5	0.5–0.6	n.r.	20	1 M NaClO_4_ in DME	1C	1018 (0.1C)	542(2700)	143
S@MoS_2_-Mo_1_/SGF	80.9	1.21–3.51	n.r.	n.r.	1 M NaClO_4_ in PC/EC (1 : 1) with 5 wt% FEC	0.1 A g^−1^	1017	505(1000)	144
Zn–N_2_/CF/S (single-atom Zn catalyst)	63	n.r.	n.r.	n.r.	1 M NaClO_4_ EC/DEC (1 : 1) with 5% FEC	0.1 A g^−1^	957	396(4000)	145
S/phos-C (phosphor-doped carbon host)	70	n.r.	n.r.	n.r.	1.0 M NaClO_4_ in EC/PC (1 : 1) with 5 wt% FEC	1C	1034 (0.1C)	480(2000)	146
S/amorphous Ni-B@GO catalyst composite	n.r.	n.r.	5.75	n.r.	1 M NaTFSI in PC/FEC	0.2 A g^−1^	1045	470(1000)	20
Te-doped sulfurized polyacrylonitrile nanoflower (FTe_0.01_S_0.99_PAN)	49.71	12.3	16.1	n.r.	NaFSI/DME/TTE (1 : 1.2 : 1)	1C	649 (0.5C)	605(2400)	147
Mn–N_2_/CNs@S (Mn single-atom catalyst on carbon nanospheres)	69.8	0.9	n.r.	n.r.	2 M NaTFSI in PC/FEC (1 : 1)	3.0C	595	458(2300)	148
S@biomass porous activated carbon (AC)	50	n.r	n.r.	30	1 M NaTFSI in EC/EMC/DMC (1 : 1 : 1) + 5 wt% FEC	0.5C	730.6	610.3(400)	149
S@Fe-Co/NC (Fe-Co diatomic sites on N-doped carbon)	50	5.6	4.36	n.r.	1.0 M NaClO_4_ in EC/DMC (1 : 1) with 5%	1 A g^−1^	593	379 (2000)	150
S@VCo DACs/N-MXene (V/Co dual-atomic sites on N-MXene)	66.7	0.8–1.2	n.r.	n.r.	1 M NaSO_3_CF_3_ in DEG/DME	1.0C	1255	1078(1500)	151
S@ZnSe/NC (ZnSe nanoparticles on N-doped carbon)	61.62	0.8–1.0	n.r.	n.r.	1 M NaClO_4_ in PC/EC (1 : 1) with FEC	0.1 A g^−1^	1565	720(100)	152
S-SPAN (sulfurized polyacrylonitrile)	46	2	n.r.	n.r.	2 M NaFSI in DMP/CPME (1 : 1)	n.r.	750	530(200)	153
LMCF@S (lignin-based microporous carbon nanofibers/S)	54	n.r.	n.r.	n.r.	1 M NaClO_4_ in EC/PC (1 : 1)	1 A g^−1^	820	642(100)	154
NMCS (N-doped microporous carbon–sulfur composite)	50	0.6–0.8	n.r.	190	3 M NaFSI in EC/PC (1 : 1)	1 A g^−1^	2193.6	752.7(200)	155
N,O-codoped corn-straw derived carbon/S (NOC/S)	40.1	n.r.	n.r.	n.r.	1 M NaCF_3_SO_3_ in diglyme	1C	325	262(1000)	156
MS-1M@S	55	n.r.	n.r.	n.r.	1 M NaClO_4_ in EC/PC (1 : 1) with 3 wt% FEC	0.1 A g^−1^	623	360(1000)	157
SPAN@AC	42.46	n.r.	n.r.	n.r.	1 M NaPF_6_ in EC/DMC (1 : 1) with 5% FEC	0.2 A g^−1^	n.r.	400(500)	158
NbO_*x*_-DCB/S	51.6	n.r	n.r.	n.r.	1 M NaCF_3_SO_3_ in DEGDME	2 A g^−1^	1513(0.1)	547(3000)	159

Table 4 shows that the performance of Na–S prototypes cannot be evaluated comprehensively using gravimetric capacity and cycling stability alone. In this context, sulfur loading is particularly important because increasing the amount of active sulfur per unit electrode area places greater demands on ionic/electronic transport and sulfur-conversion kinetics.^160^ Several recent RT Na–S studies have extended their electrochemical evaluation to higher sulfur loadings (Table 4). For example, the sulfur loading of S@MoS_2_-Mo_1_/SGF was increased from 1.21 to 3.51 mg cm^−2^, with the high-loading electrode delivering 769 mAh g^−1^ initially and retaining 530 mAh g^−1^ after 100 cycles at 0.1 A g^−1^.^144^ N-MXene@MWCNT-MP/S was evaluated at a sulfur loading of 5.5 mg cm^−2^ and 2C,^58^ while Fe–Co/NC cathodes were further evaluated at sulfur loadings of 3.2 and 5.6 mg cm^−2^ at 0.1 A g^−1^.^150^ These studies also identify high sulfur loading as an important parameter for evaluating RT Na–S batteries under conditions relevant to high-energy-density or practical applications,^58,144,150^ supporting its use in assessing whether the electrochemical advantages of a cathode design are retained as the amount of active sulfur per unit area increases. However, sulfur loading should be considered together with areal capacity and the *E*/*S* ratio.^161^ Higher sulfur loading becomes more meaningful at the cell level when the additional sulfur contributes effectively to areal capacity without requiring an excessive electrolyte inventory.^51^ Progress toward practically relevant RT Na–S cells therefore depends on a balance between sulfur loading, sulfur utilisation, areal output, electrolyte demand, and cycling stability rather than on a single performance metric. Table 4 also reveals an important limitation for cross-study comparison: sulfur loading is reported for only part of the compiled prototypes, while areal capacity and *E*/*S* ratio are provided less frequently, and cathode density is rarely disclosed. This incomplete reporting limits direct comparison of cell-level performance and highlights the need for more systematic reporting of practical cell parameters.^161^ Specifically, among the Na–S cell prototypes summarised in Table 4, cathode framework design has emerged as a critical factor influencing sulfur utilisation and long-term electrochemical performance. Two predominant strategies are evident: using porous conductive hosts that physically confine sulfur species, and sulfurised polyacrylonitrile (S-PAN)-based architectures that chemically immobilise sulfur within a polymer matrix.^56,135,153,158,162^

Porous carbon frameworks have been widely used because of their high specific surface area, good electronic conductivity, and ability to accommodate volumetric expansion.^163,164^ In such systems, sulfur is primarily stabilised through physical confinement within micro- and mesoporous structures.^157^ However, despite structural buffering, long-chain polysulfide dissolution may still occur, particularly during extended cycling.^163^ In contrast, S-PAN-based cathodes are characterised by covalent bonding between sulfur moieties and the polymer backbone, resulting in a fundamentally altered reaction pathway toward a quasi-solid-state conversion mechanism.^165,166^ This structural feature effectively suppresses the formation and dissolution of long-chain polysulfides, thereby enhancing cycling stability in fully assembled Na–S cell prototypes.^166^ However, material-level structural stabilisation alone is insufficient to ensure long-term electrode durability.

Researchers have further controlled sulfur confinement by modifying the cathode framework. In an RGO/SiO_2_/S cathode, sulfur was incorporated into porous fumed SiO_2_, followed by wrapping of the composite with an interconnected N-doped RGO network.^167^ The porous SiO_2_ accommodated sulfur and buffered volume changes, while the RGO network improved electronic and ionic transport and reduced polysulfide dissolution.^167^ In another design, KOH-activated almond-shell-derived hard carbon containing interconnected micro- and mesopores was used as the sulfur host.^168^ Sulfur was predominantly confined within the micropores, which was associated with the preferential formation of short-chain insoluble polysulfides rather than soluble long-chain species.^168^ A capacity of 736.1 mAh g^−1^ was maintained after 300 cycles at 0.2C.^168^

Beyond physical confinement, researchers have also investigated chemically and catalytically active sulfur hosts to regulate polysulfide adsorption and conversion. In Li–S systems, researchers have explored polar and catalytic host materials and heterostructured interfaces to strengthen polysulfide anchoring and promote their subsequent conversion.^169–172^ Researchers have also investigated heterointerface engineering to regulate the electronic structure of active sites. For example, the d-band centre of Co at the CoSe_2_@CoSe_0.25_S_1.75_ heterointerface was identified as a descriptor of the adsorption and conversion abilities toward lithium polysulfides.^170^ Related approaches have also been reported for RT Na–S batteries. Fe_3_N incorporated into an N-doped porous carbon network was reported to interact with sodium polysulfides through Na–N and Fe–S interactions, while sulfur accommodation and buffering of volume changes were provided by the porous carbon framework.^173^ Fe_3_N also promoted the electrochemical conversion of NaPSs.^173^ Mo_2_N-W_2_N heterostructures embedded in porous carbon have been used as sulfur hosts, with the porous framework providing physical sulfur confinement and accommodating volume expansion, while the heterostructure promotes NaPS adsorption and catalytic conversion to Na_2_S.^174^ MoC-W_2_C heterostructures confined within multichannel carbon nanofibers have also been investigated for polysulfide confinement and accelerated NaPS conversion.^174^

In practical S-PAN-based cathodes, both the sulfur composite design and the binder's functionality are recognised as critical factors governing structural and electrochemical stability. This consideration becomes particularly important in Na–S systems, where the S → Na_2_S conversion is accompanied by a severe volumetric expansion (180%), leading to substantial mechanical stress within the cathode framework.^162^ To shed light on the molecular origin of binder-dependent stabilisation, density functional theory calculations in ref. 135 examined interactions between polymer functional groups and sodium sulfide species. Stronger coordination interactions were identified between Na_2_S and the carboxyl-containing poly(acrylic acid) (PAA) compared to poly(vinylidene fluoride) (PVDF), as reflected by higher binding energies (2.02 eV *vs.* 1.20 eV), indicating enhanced interfacial affinity (Fig. 7).

**Fig. 7:**
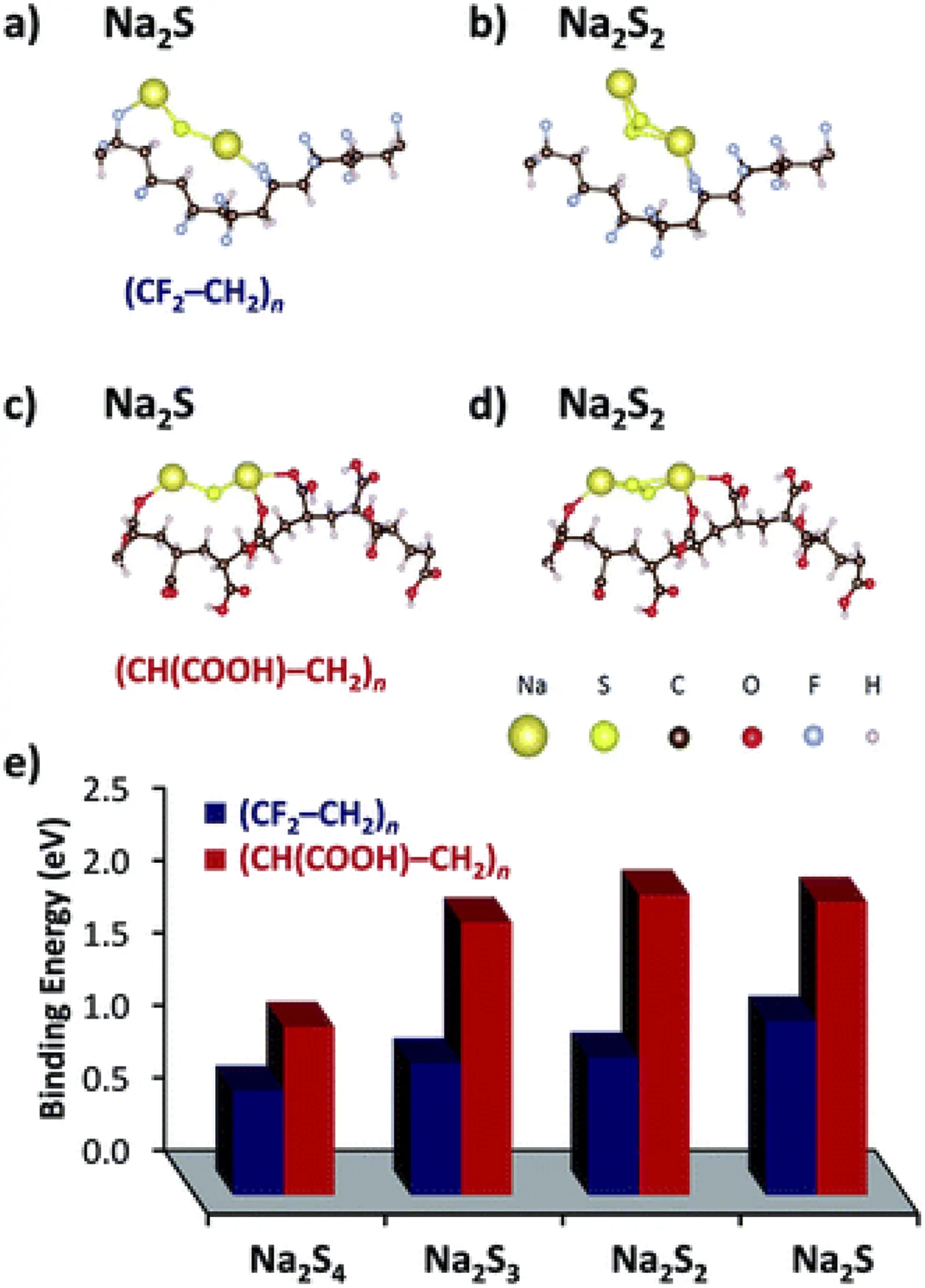
Density functional theory calculations. Optimised binding configurations of the NaSB discharge product and intermediates with (a and b) polyvinylidene difluoride (CF_2_–CH_2_)_*n*_ and (c and d) polyacrylic acid (CH(COOH)–CH_2_)_n_. Each polymer is constructed with eight repeating units in a unit cell. The polymer is repetitive along the chain axis. Binding configurations of the polymers with other short-chain sodium polysulfides, Na_2_S_3_ and Na_2_S_4_. (e) Tabulated binding energies of sodium sulfide and short-chain sodium polysulfides with the two binder structures. Reproduced from ref. 135 with permission from the Royal Society of Chemistry, copyright © 2020.

Post-mortem SEM analysis further supported these theoretical findings. Pronounced structural differences were observed between the two binder systems (Fig. 8).^135^ While PAA-based cathodes preserved morphological integrity and uniform sodium distribution, PVDF-containing electrodes showed severe cracking and locally poorly sodiated regions, suggesting insufficient mechanical stabilisation during repeated volumetric expansion.

**Fig. 8:**
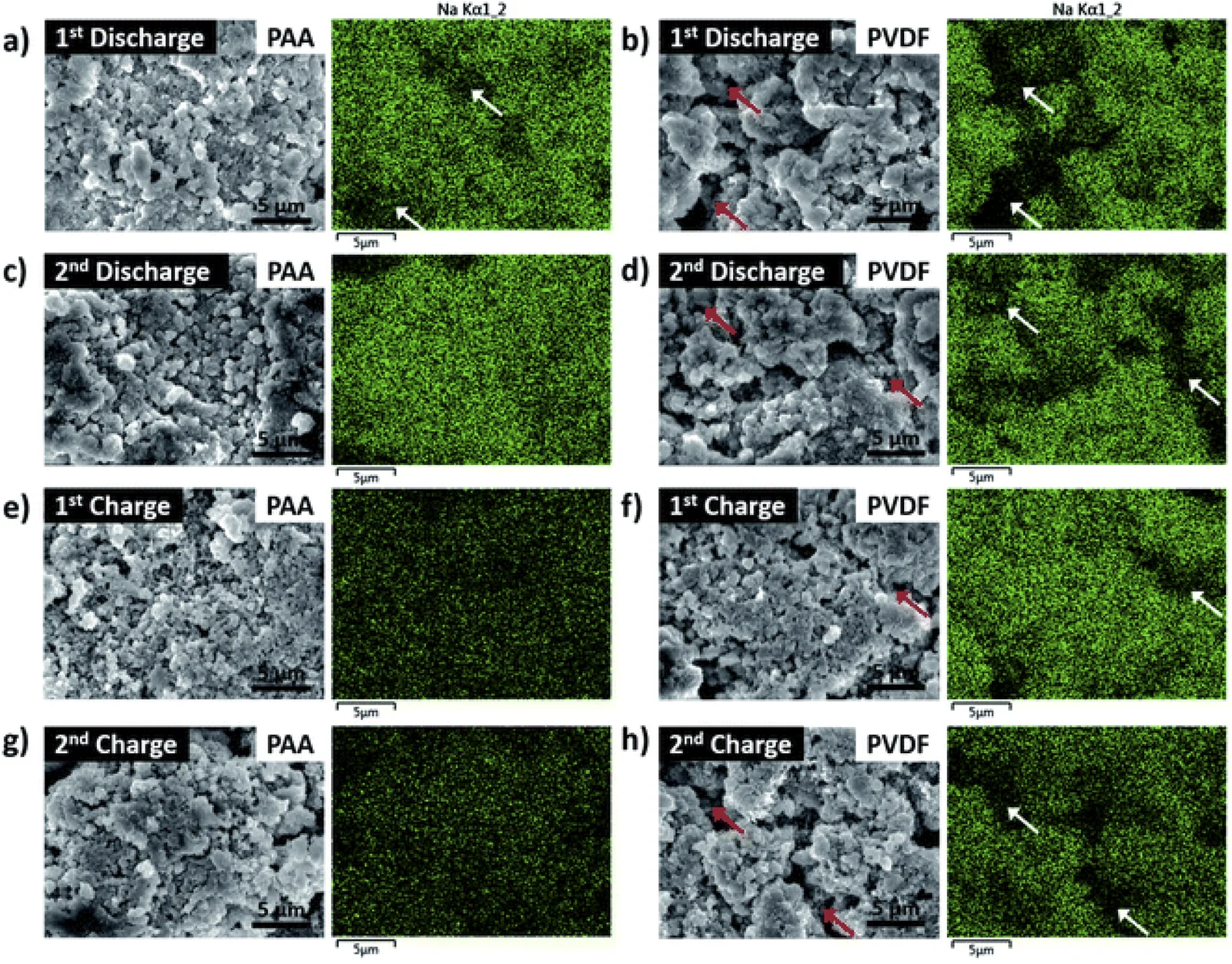
Scanning electron micrographs of cycled S-PAN cathodes and their corresponding sodium elemental maps obtained by energy dispersive X-ray spectroscopy for cathodes containing (a, c, e and g) PAA binder (left panel) and (b, d, f and h) PVDF binder (right panel). The red and white arrows indicate void spaces and regions with a lower sodium content, respectively. Reproduced from ref. 135 with permission from the Royal Society of Chemistry, copyright © 2020.

The evolution of the galvanostatic profiles further demonstrated a pronounced binder-dependent behaviour. The influence of binder chemistry became even more evident during extended cycling (Fig. 9).^135^ PAA-based cathodes maintained sustained reversible capacity and improved rate capability over hundreds of cycles, whereas PVDF systems showed rapid capacity decay and structural destabilisation. Additional analysis after prolonged cycling revealed the formation of a surface interfacial layer; the PVDF system showed more pronounced film development, whereas PAA-based cathodes maintained a more uniform interfacial structure.^135^ Collectively, these findings indicate that binder chemistry is critical to governing not only mechanical integrity but also long-term electrochemical reversibility in S-PAN-based Na–S batteries.

**Fig. 9:**
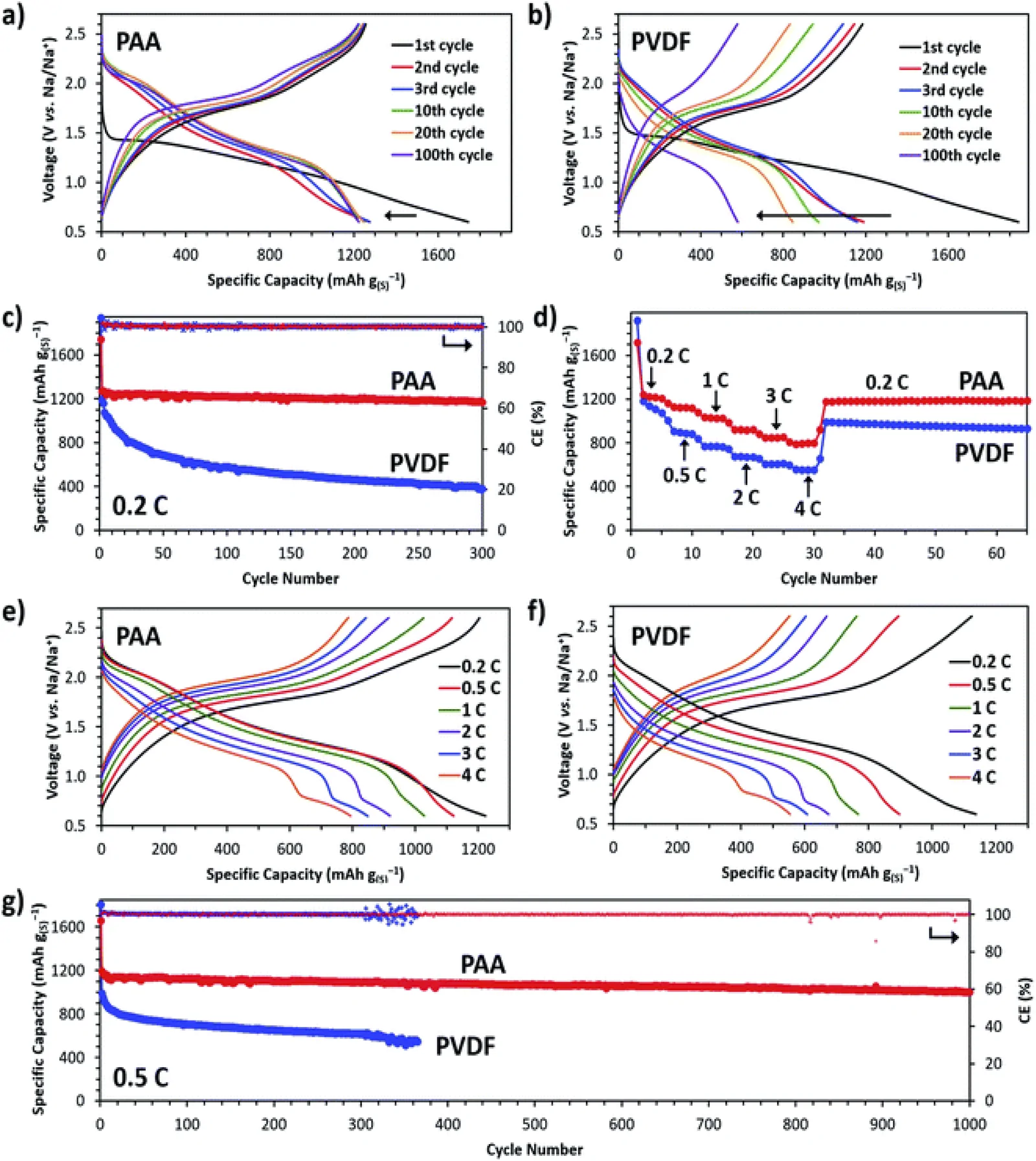
Galvanostatic charge/discharge profiles for sodium–sulfur cells at 0.2C, containing S-PAN cathodes with (a) PAA *vs.* (b) PVDF binders. (c) Cycle performance and coulombic efficiencies of sodium–sulfur cells at 0.2C. (d) Rate performance comparison and galvanostatic charge/discharge profiles for (e) PAA-based and (f) PVDF-based cathodes between 0.2C and 4C, where 1C = 1673 mA g (S)^−1^. (g) Extended galvanostatic cycling at 0.5C for S-PAN Na–S cells. Reproduced from ref. 135 with permission from the Royal Society of Chemistry, copyright © 2020.

Building on this understanding, the influence of binder chemistry in another prototype configuration integrating S-PAN with Ti_3_C_2_T_*x*_ MXene.^166^ In this hybrid conductive architecture, electrochemical performance was governed not only by the strength of Na–O coordination but also by polymer topology and interfacial compatibility with the MXene surface. Improved dispersion of S-PAN and enhanced polysulfide anchoring were achieved with carboxymethyl cellulose (CMC), resulting in superior cycling stability and rate capability relative to alternative binder systems. Beyond conventional cathode design strategies, such as host-structure engineering and binder optimisation, external magnetic fields have recently attracted attention as a promising non-invasive approach to improve Na^+^ transport and mitigate polysulfide shuttling in Na–S batteries.^175^

## 7 Positioning Na–S batteries within the family of metal–sulfur energy storage systems

Given the promising characteristics of Na–S systems, including their high theoretical energy density, low cost, and abundant raw material availability, researchers commonly evaluate their performance in a broader context by comparing them with other M–S battery technologies. M–S systems are widely regarded as one of the most important directions in energy storage research owing to the unique combination of sulfur's high theoretical energy density, natural abundance, and environmental benignity. Nevertheless, the efficiency, stability, and practical feasibility of M–S batteries depend primarily on the choice of metal anode. In terms of gravimetric and volumetric energy density, Li–S batteries have some of the highest theoretical values, reaching 2612 Wh kg^−1^ and 2955 Wh L^−1^, respectively (Table 5). Despite these advantages, the sustainable scalability of Li–S systems is constrained by lithium's limited natural abundance, estimated at approximately 20 ppm in the Earth's crust and 0.000018% in seawater.^176^ In contrast, Na–S batteries exhibit moderate theoretical energy densities (1270 Wh kg^−1^ and 1545 Wh L^−1^); however, the exceptional abundance of sodium—approximately 23 600 ppm in the Earth's crust and 1.1% in oceans—renders Na–S systems particularly suitable for stationary and large-scale energy storage applications.^9^

**Table 5:** Comparison of sodium and other metal anodes for M–S battery applications (prices as of September 2025, Sigma-Aldrich)

Element	Theoretical specific energy, Wh kg^−1^	Volumetric capacity, mAh (cm^−3^)^−1^	Reversibility	SEI stability	Density, g (cm^3^)^−1^ (ref. 177)	Crustal abundance in ppm and % (ref. 177)	Price in € (ref. 178)
Li	2612	2045	High	High	0.535	20, 0.0017	297.00(100 g)
Na	1270	1128	High^179^	High^179^	0.968	23 600, 2.3	611.00(500 g)
							167.00(100 g)
K	916	612	Low^180^	Low^180^	0.89	20 900, 5%	153.00(50 g)
Ca	1838	2064	Low^181^	Low^181^	1.55	41 500, 5%	126.00(100 g)
							497.00(500 g)
Mg	2300	3882	Moderate^182^	Moderate^182^	1.738	950, 2.9%	98.80(250 g)
							248.00(1 g)
Zn	2372	n.r.	Moderate^183^	Moderate^183^	7.14	750.0078%	227.00(10 g)
							759.00(50 g)
Cu	547	n.r.	Low^184^	Low^184^	8.96	500.0068%	296.00(10 g)
							1140.00(50 g)
S	1675	n.r.	n.r.	n.r.	1.96	0.042%	89.60(50 g)
							327.00(250 g)

Compared to K–S batteries, sodium-based systems demonstrate more favourable overall electrochemical characteristics. Although potassium has a theoretical energy density of 916 Wh kg^−1^ (Table 5), K–S batteries are significantly affected by substantial volume expansion up to 309% and the formation of unstable solid electrolyte interphase (SEI) layers. These factors reduce cycling stability and accelerate anode degradation. Similar limitations are observed in calcium–sulfur (Ca–S) batteries, where elevated SEI impedance impairs reversible electrochemical reactions and limits sulfur utilisation to approximately 35%. M–S systems employing zinc and copper have garnered interest due to their high theoretical energy densities of 2372 Wh kg^−1^ and 547 Wh kg^−1^, respectively. Nonetheless, their practical gravimetric performance is hindered by the high density of these metals, and their limited natural abundance – 75 ppm for zinc and 50 ppm for copper -presents additional challenges for large-scale application, as detailed in Table 5. Moreover, copper–sulfur (Cu–S) batteries suffer from dendritic copper growth, requiring sophisticated anode engineering strategies to maintain operational stability and safety. Based on the comparative metrics summarised in Table 5, sodium emerges as the most balanced metallic component among M–S battery systems. Its widespread consideration stems from its remarkable natural abundance, acceptable energy density, and relatively improved interfacial stability. These advantages make Na–S batteries increasingly recognised as promising candidates for sustainable, large-scale energy storage. Building on sodium's favourable characteristics as an anode material, sulfur is often used as the cathode in Na–S batteries because of its high theoretical capacity (1675 mAh g^−1^), low cost, and broad availability. However, sulfur exhibits inherently low electrical conductivity^31^ and tends to dissolve intermediate polysulfide species into electrolytes—a prevalent challenge across all M–S battery systems. Compared to other M–S chemistries, where sulfur-related issues are compounded by fundamental anode instability, sluggish interfacial kinetics, or significant volume changes, Na–S batteries offer a more balanced profile across energy characteristics, resource availability, and interfacial stability. Therefore, Na–S systems are a rational and potentially practical alternative to Li–S technology for sustainable, large-scale energy storage.

## 8 Conclusion and perspectives

Room-temperature sodium–sulfur (RT Na–S) batteries are a promising, low-cost, high-energy-density alternative to lithium-based chemistries, yet they still suffer from polysulfide dissolution, the shuttle effect, kinetic and mass-transport limitations, and interphase degradation, which collectively hinder practical deployment. A key challenge in overcoming these physical constraints is the limited availability of systematic, reliable data mapping NaPS transport behaviour across complex electrolyte–host interfaces. To move beyond generic performance optimisations, future research should prioritise the following: first, advanced (localised) high-concentration liquid, solid (quasi-solid) electrolytes, and emerging high-entropy electrolytic platforms to suppress polysulfide dissolution and passivate the reactive Na anode. Secondly, implementing functional separators modified with electrocatalytic sites to block polysulfide crossover and accelerate the conversion kinetics of insoluble NaPSs. Another priority is mechanical and electrochemical interface stabilisation: designing multifunctional conductive hosts to tailor the accommodated sulfur volume while mitigating non-uniform sodium dendrite growth. To better understand the mechanisms and track dynamic sulfur electrochemistry, advanced experimental techniques such as *in situ* investigation and *operando* approaches are needed.

Applying machine learning platforms and computational workflows to address these issues remains an early-stage, rapidly evolving field. However, direct ML/AI studies specifically focused on RT Na–S batteries remain scarce, and most current approaches are extrapolated from related battery chemistries. Together with ML- and AI-driven battery health diagnostics^185,187^ and lifetime prediction,^188^ these neural network-based tools, as already reported, can optimise battery performance^189^ and discover new electrode materials,^186^ including high-performance electrolytes and separators. Future research on RT Na–S should also focus on three core ML- and AI-integrated research areas: (1) machine learning for solvation engineering, (2) AI-assisted catalytic interlayer design, and (3) standardised *in situ* model validation.

## Author contributions

Akbota Kelgenbayeva: conceptualization, writing an original draft, data curation, visualization. Aitolkyn Uali: conceptualization, data curation, supervision, writing an original draft, visualization. Zhumabay Bakenov: conceptualization, supervision. Arailym Nurpeissova: writing – review & editing, validation, supervision, project administration, funding acquisition, conceptualization. Aliya Mukanova: writing – review and editing, writing – original draft, validation, supervision, project administration, conceptualization.

## Conflicts of interest

No potential conflict of interest.

## Data Availability

This review article is based on previously published literature. All data discussed in this review are derived from previously published studies cited in the reference list. No new datasets were generated for this study.
